# Planning and optimizing a digital self‐management support intervention: Acne Care Online

**DOI:** 10.1111/bjhp.70033

**Published:** 2025-11-05

**Authors:** Rosie Essery, Mary Steele, Stephanie Easton, Sebastien Pollet, Charlotte Cairns, Rebekah LeFeuvre, Julie Hooper, Taeko Becque, Tanith Kane, Georgina Hart, Tracey Sach, Nick Francis, Paul Little, Lucy Yardley, Sophie Dove, Kate Henaghan‐Sykes, Irene Soulsby, Kim S. Thomas, Matthew J. Ridd, Beth Stuart, Alison M. Layton, Andrew R. Thompson, Mahendra Patel, Adam Yates, Miriam Santer, Ingrid Muller

**Affiliations:** ^1^ University of Southampton Southampton UK; ^2^ University of Bristol Bristol UK; ^3^ Public Contributor UK; ^4^ University of Nottingham Nottingham UK; ^5^ Queen Mary University of London London UK; ^6^ Skin Research Centre, Hull York Medical School University of York York UK; ^7^ Cardiff and Vale University Health Board Cardiff University Cardiff UK; ^8^ Oxford University Oxford UK; ^9^ Woodstock Bower Group Practice Rotherham UK

**Keywords:** acne vulgaris, behaviour change, digital intervention, intervention development

## Abstract

**Objectives:**

To showcase the planning and optimization processes involved in developing a digital behaviour change intervention through the example of a self‐management support tool for young people with acne (‘Acne Care Online’).

**Design:**

Following Medical Research Council guidance, a theory, evidence, and person‐based approach was employed, drawing on existing evidence, stakeholder expertise, health psychology theory, and qualitative methods to underpin intervention content, structure and functionality.

**Methods:**

Systematic reviews of literature concerning acne help‐seeking and treatment adherence, theoretical understandings of health‐related behaviour, guidance from public contributors, and interviews with young people with acne (*n* = 24), their parents/carers (*n* = 8) and healthcare professionals (*n* = 18), informed the intervention's guiding principles and logic model. Draft intervention content was then developed by a multidisciplinary study team including public contributors and health professionals, and optimized through 53 think‐aloud interviews with intended users.

**Results:**

The development process created Acne Care Online ready for trial evaluation. It also provided insights into self‐management challenges amongst this group – including their reluctance to consult, and misconceptions about treatments that hinder effective management (e.g., using products with no active ingredients, concerns about side effects, and unrealistic expectations). Acne Care Online appeared engaging, informative and relevant, with early feedback from health professionals suggesting it could be integrated into current healthcare practice.

**Conclusions:**

This study provides insights into theory and person‐informed development processes for behaviour change interventions. Here, the acceptability and perceived value of Acne Care Online was evidenced. The work also provides important insights for clinicians managing young people seeking support for acne.

**Statement of Contribution**

**What is already known on this subject?**

- Acne is very common, significantly impacting individuals' quality of life and contributing to substantial healthcare resource use.
- Topical acne treatments are very effective when used appropriately, but are often not well used or understood. Instead there is an over‐reliance on non‐evidence based ‘off the shelf’ products, and in some cases, potentially avoidable long‐term antibiotic treatment with implications for antimicrobial resistance.
- Accessible and engaging self‐management support for young people with acne is needed to increase effective use of topical treatments with a view to improving acne‐related outcomes, and reducing unnecessary antibiotic treatment of acne.

**What does this study add?**

- A systematic report of developing theory, evidence and person‐based digital support for young people with acne.
- Comprehensive analysis of what influences young people's help‐seeking and treatment adherence behaviour for acne.
- Key considerations for clinicians for providing effective and sensitive care in consultations for acne.

## INTRODUCTION

Acne vulgaris (acne) is very common, affecting an estimated 9.4% of the world's population (Layton et al., 2021). Up to 95% of individuals are likely to be affected in their lifetime (Heng & Chew, 2020). Experiencing acne is commonly associated with poorer emotional wellbeing and quality of life (Tan et al., 2021; Williams et al., 2012), increased risks for depression and anxiety (Samuels et al., 2020), and may increase the risk of suicidal ideation and/or behaviours amongst adolescents (Barlow et al., 2023). In addition to its impact on individuals, acne management places significant demand on healthcare services. Estimates of annual UK primary care consultations for acne range from close to one million (Francis et al., 2017) to 3.5 million (Wilcock et al., 2021). Whilst estimates vary, there is a widespread unmet need, with many more people with acne not consulting in primary care (Francis et al., 2017; Wilcock et al., 2021). Acne also contributes substantially to long‐term antibiotic use amongst young people. In the UK, the median duration of oral antibiotic use for acne is 8.5 months (Bhate et al., 2022), with increasing concern that this contributes to antimicrobial resistance (AMR; Bhate et al., 2022; Walsh et al., 2016). There is a need for effective, early treatment of acne to improve outcomes for individuals and to reduce unnecessary antibiotic treatment.

Topical treatments (i.e., creams, gels, facewashes) can be highly effective in treating most mild–moderate acne (Eichenfield et al., 2021; Leung et al., 2021; Stuart et al., 2021). Combination therapies involving topicals (e.g., using a topical treatment alongside an antibiotic) are often more effective than antibiotic treatment alone and have the potential to reduce the duration of antibiotic treatment (Bienenfeld et al., 2017; Corcoran et al., 2023). All international guidelines recommend topical treatments amongst first‐line options for mild–moderate acne (Corcoran et al., 2023). Despite this, the use of such treatments amongst young people with acne is suboptimal for several reasons (Dréno et al., 2010). First, young people are often poorly informed about topical treatments, instead commonly relying on widely marketed and readily available dermo‐cosmetic products (Araviiskaia et al., 2022; Ip et al., 2020). Many of these products do not contain sufficient, if any, quantities of the evidence‐based medications to actively treat acne, and are often expensive (Goff & Stein, 2025). Whilst a small number of topical treatments can be purchased ‘off‐the‐shelf’ in the UK, the majority are sold under the supervision of a pharmacist, or are prescription‐only medications (National Health Service, 2025).

Secondly, amongst those who *do* access evidence‐based topical treatments, many do not use them as directed, nor regularly enough, to achieve benefit (Dréno et al., 2010). Common reasons for suboptimal adherence include unrealistic expectations about treatments' onset of action, and side effects such as skin dryness and flakiness (Araviiskaia et al., 2022). These often result in people stopping regular application before the treatment has had sufficient time to have an effect, because of discomfort or belief that it is not working (Araviiskaia et al., 2022; Gollnick et al., 2016). There is a need to understand these help‐seeking and adherence behaviours and their determinants – theoretical models of health psychology and behaviour change are a vital part of this understanding. Simple education and management strategies could be beneficial for adherence and outcomes in dermatological treatment including amongst young people with mild, moderate, and severe acne (Donnarumma et al., 2019; Marasca et al., 2020; Thiboutot et al., 2008). There is preliminary evidence for the acceptability and feasibility of interventions focused on changing acne‐related behaviours by communicating credible information and enhancing perceived control in the long‐term management of acne through addressing misinformation and optimizing beliefs and knowledge about acne and appropriate treatments (Ip, Muller, Geraghty, Platt, et al., 2021; Ip, Muller, Geraghty, Rumsby, et al., 2021). Further support for young people with acne to understand, access, and adhere to evidence‐based treatments is needed.

Digitally delivered behaviour change interventions have increasingly been established as an effective, broadly accessible, and cost‐effective means of delivering self‐management support for a range of health conditions (Michie et al., 2017). A digital approach to acne self‐management support seems especially appropriate given indications that telehealth approaches have shown promise in improving adherence to acne treatments (Donnarumma et al., 2019; Marasca et al., 2020). Furthermore, young people aged 16–24 are the most frequent users of the internet (Office for National Statistics, 2021) and report high levels of engagement with online health‐related information and advice seeking (Park & Kwon, 2018). Amongst other common dermatological conditions, recent research has provided evidence of the effectiveness, cost‐effectiveness and acceptability of digital self‐management support for eczema amongst young people and their parents (Sach et al., 2024; Santer et al., 2022). The process evaluation of Eczema Care Online (ECO) revealed that even relatively limited online engagement was sufficient to achieve an improvement in outcomes (Greenwell et al., 2022, 2024). On this basis, a digital behaviour change intervention seems a potentially promising means of supporting young people to understand, access, and adhere to evidence‐based acne treatments. Although small studies have attempted to provide support for acne self‐management (Ip, Muller, Geraghty, Rumsby, et al., 2021; Wang et al., 2015), to date, no comprehensive resource exists.

To develop such an intervention, Medical Research Council (MRC) guidance advocates a theory‐, evidence‐ and person‐informed approach (Skivington et al., 2021). This facilitates identification of the most appropriate target behaviours, likely determinants of those behaviours and how to address these, and ensures that specific needs, preferences, and life contexts of target users are accounted for (Kok & Schaalma, 2004; Skivington et al., 2021; Yardley et al., 2015). In line with this best practice guidance, the aims of this work were: to apply a theory‐, evidence‐, and person‐informed approach to developing the ‘Acne Care Online’ digital behaviour change intervention to support self‐management of acne amongst teenagers and young adults.

In doing so, two secondary aims of this work were: to explore acne self‐management support needs amongst young people; and to provide evidence about the acceptability, persuasiveness and relevance of digitally delivered self‐management support amongst young people with acne. The aim of this article is to document this development process and its key findings. Clear reporting avoids duplication of ineffective, unfeasible, or unacceptable interventions (O'Cathain et al., 2019).

## MATERIALS AND METHODS

We employed the person‐based approach (PBA; Yardley et al., 2015) to develop Acne Care Online. The PBA provides a systematic framework for developing complex behaviour change interventions. It facilitates the collation of the contextually embedded experiences and perspectives of the intervention's intended users with relevant empirical evidence and behaviour change theory. This can then underpin the development of an intervention's content, structure and functionality (Yardley et al., 2015).

**FIGURE 1:**
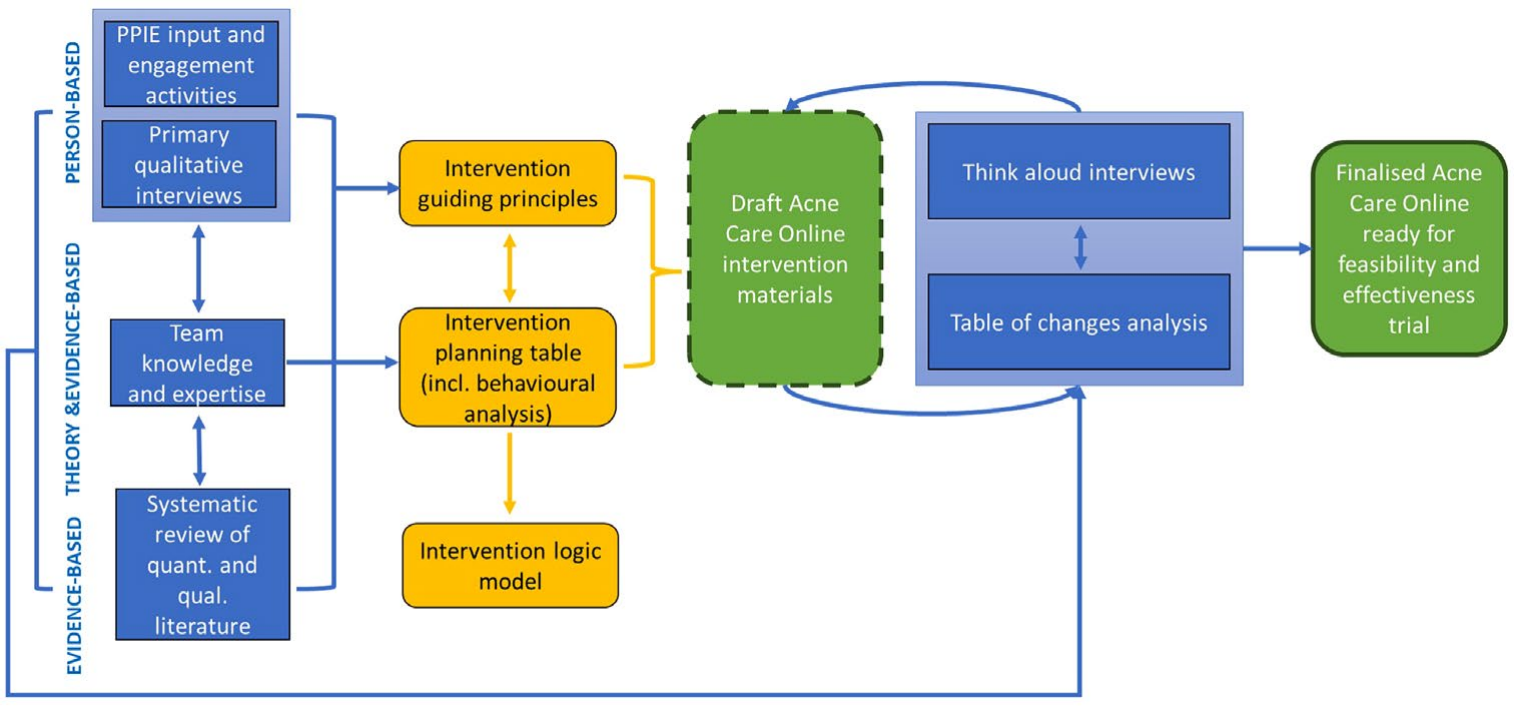
Overview of the planning and optimization processes involved in developing Acne Care Online.

Using this approach, the development of Acne Care Online was implemented in two broad phases using multiple methods: (1) Planning, and (2) Optimization, illustrated in Figure 1, and described below.

### Planning

Four main planning phase activities contributed to the theory, evidence and person‐based insights that underpin Acne Care Online content, structure and functionality. These were (1) qualitative interviews with young people aged 13–25 years with acne, their parents, and healthcare professionals (HCPs); (2) systematic reviews of the quantitative and qualitative literature about young people's help‐seeking and adherence‐related behaviours around acne; (3) extensive Patient and Public Involvement and Engagement (PPIE) activities; and (4) multidisciplinary study team knowledge and expertise. The specific methods involved in each of these activities are outlined below. Details of the qualitative interviews, systematic review and PPIE work are all described more fully in forthcoming publications, so are only summarized here.

#### Qualitative interviews

The aims of the qualitative interviews were to provide an in‐depth understanding of the experiences of young people and their parents/carers in managing acne. This enabled the identification of the most salient self‐management support needs amongst this group and facilitated understandings of how the intervention could be made engaging, persuasive and relevant. We also sought to understand HCPs' experiences of treating acne to ensure that considerations about how Acne Care Online may be implemented and disseminated within existing healthcare systems were considered from the earliest stage.

##### Recruitment

Young people aged between 13 and 25 with current or historic experience of acne (*n* = 24), and the parents or carers of some of those aged 13–15 (*n* = 8) were recruited from across England via Facebook and Instagram social media advertising. We took a maximum variation approach to sampling, aiming to achieve a diverse sample of individuals reflective of characteristics such as age (mean = 16.04 years), gender (71% girls/young women, 25% boys/young men, 4% prefer not to say), ethnicity (54% from Asian, Black or ‘Other’ ethnic backgrounds), acne severity and duration, and relative deprivation status indicated by indices of multiple deprivation (IMD). We were especially keen to ensure our sample represented the views of younger teenagers (under 16), those identifying as male, and those from Black and south Asian ethnic minority backgrounds. This was due to the prior under‐representation of these groups in qualitative research on this topic, and the recognition that there may be a need for specialized approaches to the management of skin conditions in people with diverse skin tones (Alexis et al., 2021; Ip, Muller, Geraghty, Platt, et al., 2021).

HCPs were identified via primary care practices engaged in the wider study, re‐contact of HCPs who had recently participated in another study, and via advertising in a closed social media group for Allied Health Professionals. We recruited a diverse sample of HCPs (*n* = 18) in terms of their professional role and experience, including GPs (*n* = 7), nurse prescribers (*n* = 3), paramedic prescribers (*n* = 3) and community and practice pharmacists (*n* = 5).

##### Procedure

On seeing the social media advert (young people or their parent/carer) or receiving an email invitation (HCPs), interested recipients followed a link to the study website where they could find additional information, and complete an expression of interest form including basic demographic details. For under 16‐year‐olds, social media advertising was via their parent or carer in the first instance. After completing an online consent form for their teenager to be invited to participate, a parent/carer could indicate whether they wished to take part in an interview themselves. After receiving an expression of interest, the study team contacted the individual to arrange a convenient time for the interview and to share the link to the online consent form to be completed prior to the interview. For those under 16, an online assent form was completed in addition to a parental consent form.

Each person took part in one semi‐structured qualitative interview conducted over the phone or via Microsoft Teams. Where a young person *and* their parent were interviewed, these were either conducted jointly, or consecutively depending on the preference of the young person and their parent. Interviews were audio‐recorded and transcribed.

##### Analysis

Data were analysed inductively guided by the principles of reflexive thematic analysis (Braun & Clarke, 2006) to allow deep understanding and interpretation of the participants' experiences to inform intervention planning.

#### Systematic reviews

The systematic reviews aimed to provide insight into existing evidence about acne‐related adherence and help‐seeking behaviours, including their determinants and consequences, to inform the most appropriate targets and strategies for intervention. This involved both updating our team's previous qualitative review of the literature (Ip, Muller, Geraghty, Platt, et al., 2021) and adding a review of the quantitative literature on this topic. Five databases (PubMed, Medline, CINAHL, Psychinfo, and Embase) were searched according to our predefined search strategy (PROSPERO). Records were screened, and eligible studies were quality assessed and relevant data extracted. This resulted in an additional nine qualitative papers to update the existing review, and 107 quantitative studies including 10 randomized controlled trials (RCTs), and 97 observational, cohort, and descriptive cross‐sectional studies.

#### PPIE

The PPIE activities collectively aimed to complement the qualitative work in ensuring a broad range of views and experiences informed how Acne Care Online could be made as accessible, relevant and engaging as possible. Whilst the qualitative work gave insight into these issues, those individuals who have the time, inclination, confidence and capacity to engage in participatory qualitative research may not always be wholly characteristic of the wider group that they represent. Accordingly, it is important to utilize alternative and additional means of engagement with a more diverse group of intended users (Muller et al., 2019; Russell et al., 2019).

A range of PPIE input was sought in developing Acne Care Online:

- Three core public contributors – a social media content creator documenting her acne journey, a parent of teenage girls with acne, and someone with prior experience of severe acne – formed part of our intervention development group contributing regularly to discussion and decisions on all aspects of intervention development.
- An advisory panel (*n* = 24) comprising a diverse group of young people (13–22 years) with acne, including 12 White British, 4 African, 4 Indian, 2 Bangladeshi, 1 mixed White and Asian background, and 1 ‘other’ ethnicity. This group fed back on various aspects of intervention development through remote methods such as online discussion group or one‐to‐one meetings, electronic feedback on documents and online questionnaires and surveys.
- Face‐to‐face engagement activities with two schools in which we sought input on intervention content and functionality and how to involve and engage diverse groups of young people in the study.
- Face‐to‐face discussions with two established PPIE groups, each involving 8–12 young people, about recruiting young people into research studies, and input on specific elements of intervention content.

#### Study team knowledge and expertise

Our multidisciplinary study team included public contributors, clinicians, and academics providing expertise in the lived experience of acne, dermatology, primary care, clinical psychology, behavioural science and intervention development, clinical trials, statistics, and health economics. Drawing on this diverse expertise helped to identify the appropriate behaviours for the Acne Care Online intervention to target, and the most promising ways in which to do this. Input from this group mainly occurred via weekly meetings of the core study team, and approximately quarterly meetings of the wider intervention development group.

Key insights from this activity included identifying the most accurate and up‐to‐date clinical advice and evidence, and identifying potentially relevant health psychology theory and models of behaviour change to inform the development of intervention content. These included the common sense model of illness representation (CSM; Diefenbach & Leventhal, 1996), the necessity‐concerns framework (NCF; Horne, 1997; Horne, 2012; Horne & Weinman, 1999) and self‐efficacy theory (Bandura, 1977). These particular models were deemed especially relevant given their utility for theorizing how patients' beliefs about their symptoms and treatment relate to self‐management behaviours in general (Hagger & Orbell, 2003) and adherence to medications specifically (Horne et al., 2013). Self‐efficacy theory was also considered especially pertinent given its consistency in predicting health‐related behaviours and outcomes across a range of conditions (Ewulu et al., 2024; Holden, 1992) and that it makes specific recommendations for enhancing self‐efficacy (e.g., modelling, persuasion) thus helping to identify potentially appropriate intervention functions and components.

In addition to the relevant theory identified in the planning phase, we also mapped collated evidence onto constructs from the Behaviour Change Wheel (BCW; Michie et al., 2011) and Theoretical Domains Framework (TDF; Cane et al., 2012). This facilitated identification of other potentially useful behavioural determinants to target, and additional intervention functions that may maximize the intervention's effects on its target behaviours. We subsequently identified potentially appropriate behaviour change techniques using the Behaviour Change Technique (BCT) Taxonomy v1 (Michie et al., 2013).

### Optimization

The optimization phase commenced as soon as initial materials were drafted. During this phase we sought feedback through qualitative ‘think aloud’ interviews on draft materials, exploring the acceptability and persuasiveness of draft intervention content amongst target users, and iteratively refining this. In think‐aloud interviews participants are encouraged to vocalize their thoughts and feelings towards the content they are viewing, in this case allowing insight into target users' immediate reactions to the intervention and its key messages (Van den Haak et al., 2007). Although described separately here, the optimization process occurred alongside planning activities, allowing us to return to, and refine, the planning phase outputs.

#### Recruitment

Young people aged 13–25 with current or recent acne were recruited from primary care search and mailout in eight GP practices in the South of England, via targeted Facebook and Instagram social media advertising from anywhere in the UK, and through re‐sampling of planning phase interview participants. As with the planning phase, we sought to include a maximum variation sample in terms of participants' age, gender, ethnicity, acne duration and severity, and level of relative deprivation.

Participants recruited via primary care received a postal invitation from their GP practice including a participant information sheet and a brief summary flyer with a QR code linking to the study website where they could get more information or express interest. Participants recruited via social media or resampled from the planning phase interviews could follow a link (embedded in the advert or email from the study team) directly to the study website. As with the earlier interviews, those under 16 were contacted via their parent or carer initially, who completed an online consent form for their teenager to be invited.

#### Procedure

Prospective participants completed an online expression of interest form including basic demographic details, and the study team contacted them to arrange a time for the interview and to share the link to the online consent form for completion prior to the interview. For those under 16, an online assent form was completed in addition to parental consent.

Each participant took part in one ‘think‐aloud’ interview in which they worked through the draft Acne Care Online materials with an interviewer. Following the think‐aloud element, there were semi‐structured interview questions about participants' general views of Acne Care Online: what they liked/disliked, found helpful/difficult, would like to change. In later phases, we conducted six retrospective interviews whereby participants were provided with Acne Care Online a week in advance of their interview and encouraged to use it during that time, and to document any thoughts, concerns or suggestions. This allowed greater exploration of how Acne Care Online may be engaged with in a more ‘real life’ situation and a chance to obtain feedback on participants' initial experiences of implementing some of the advice or behaviour changes recommended. All interviews were audio‐recorded and transcribed.

#### Analysis

These data were analysed with the primary purpose of understanding potential changes required to optimize Acne Care Online. We collated all positive and negative comments pertaining to specific intervention elements into a ‘table of changes’ (Yardley et al., 2015). Following discussion of the frequency and significance of positive and negative comments within the study team, we coded the importance of potential changes dependent on whether an amendment was deemed likely to enhance the persuasiveness, acceptability or likelihood of changing behaviour (Bradbury et al., 2018). Changes relevant to preventing disengagement, or maximizing the chance of adhering to target behaviours were prioritized. We continued to conduct think‐aloud interviews alongside analysis to allow iterative modification of content prior to the next interviews. Once it seemed that no further important changes were required, we considered the intervention content to be sufficiently optimized (Bradbury et al., 2018).

## RESULTS

### Planning

The results of the planning phase activities comprised three distinct but interrelated outputs that guided the subsequent development of intervention materials: the intervention guiding principles, behavioural analysis and logic model, each presented below.

#### Guiding principles

Guiding principles are a core element of the person‐based approach to intervention development. They aim to ensure that an intervention is engaging, relevant and persuasive for target users by drawing on a deep understanding of their life contexts, needs and preferences. Each guiding principle comprises: (1) a design objective outlining a behavioural need, issue, or challenge specific to users and their context; and (2) intervention features that address the design objective (Yardley et al., 2015). Our guiding principles for Acne Care Online are outlined in Table 1 below with an illustration of how they were informed by all four aspects of our planning phase activities, and iteratively refined based on insights from the optimization phase.

**TABLE 1:** Acne Care Online guiding principles.

Evidence and source	Design objective	Key intervention features
Concerns about acne not warranting medical attention or ‘wasting clinicians time’ a barrier to consulting amongst YP and often parents (QI, SR, ST) Beliefs about causes/triggers of acne very varied and not always accurate (QI, SR, ST) Belief that symptoms are unpredictable/uncontrollable and something they will ‘grow out of’ (QI, SR)	To inform and manage expectations about acne symptoms and need for treatment/management	Accessible, brief (including audiovisual) information about causes of acne and how treatments work ‘Myth‐busting’ information addressing common misconceptions about acne ‘Acne Stories’ demonstrating varied experiences from diverse range of young people Advice about treatment route options (community pharmacy, primary care, online pharmacy) Balanced messaging throughout between reassurance (i.e., that acne very common and often improves with age) vs. importance of early treatment where necessary (i.e., if causing distress and to avoid possible scarring)
Prior negative experience of consultation with HCPs –didn't feel listened to, treatments prescribed were unsuccessful (QI, SR, PPIE) Many young people express preference for ‘product review’ format in making decisions about what to try (QI, PPIE) Over‐reliance on ‘off the shelf’ products that do not contain active ingredients to treat acne (SR, QI) Recognition of the negative impact on their mental health – including self‐confidence, feelings of isolation (QI, SR, PPIE) Feeling of lack of control/unpredictable course of symptoms and treatment (QI, SR) Amongst younger teenagers, parent is often gatekeeper to help and treatment‐seeking – making appointments, research, buying treatments, encouragement (QI)	To enable and facilitate control over acne journey through: Increasing knowledge about effective treatment options Increasing confidence in seeking medical advice Facilitating productive consultation/discussion with HCPs	Discussion aid tool to facilitate discussion of tailored symptom and treatment history information with HCPs Searchable ‘Product review’ tool providing key details about all evidence‐based treatments Tips and advice for what to say/what information to take to a consultation Instruction and advice about how and where to consult Messaging from HCPs (including audiovisual) that consultation for acne is important and encouraged Advice to share with parents/carers to address common barriers to consulting
Prior negative experience of treatments – treatments caused side effects or didn't seem to work (QI, SR, PPIE) Many young people rely on reviews of ‘products’ from others (usually online) in making decisions about what to try (QI, PPIE) Misunderstanding/lack of awareness that many ‘off the shelf’ products do not contain active ingredients to treat acne (SR, QI)	To inform and manage expectations about treatments in order to: Reduce barriers to appropriate use of effective treatments Reduce reliance on non‐evidence‐based products/strategies	Accessible advice about the difference between evidence‐based treatments and skincare/cosmetic products Emphasis on the need for consistent use of treatments for up to 12 weeks ‘Progress challenge’ tool to allow action planning for treatment use, encourage self‐monitoring, and providing tailored weekly feedback Tips and strategies about managing/avoiding common side effects
Acne/body positivity/neutrality a growing movement – especially driven by social media – promotes acceptance and appreciation of skin/appearance as it is without needing to be something else/better/more (QI) Recognized detrimental impact on self‐confidence, self‐esteem, loneliness and isolation, and link to social avoidance which exacerbates these (QI, SR, ST, PPIE) Lack of confidence in ability to manage impact of acne (QI, SR)	To promote reduction of physical and/or emotional discomfort as key motivation for seeking/using appropriate treatment	Overarching intervention message to encourage taking action to treat acne out of desire to address discomfort and/or distress, rather than from a need for skin to look a certain way. Strategies for managing, and signposting to additional support for managing mental health impact of acne – including confidence building, self‐compassion and managing interactions with others ‘Acne Stories’ to include advice about/experiences of living with, and acceptance of, acne
Mobile devices increasingly used instead of/alongside computers to access websites (QI, PPIE) Want key (perceived relevant) information to be available quickly without having to look through lots of pages (QI, PPIE) General preference for very brief text and short videos (PPIE, QI) Digital tools could be easily linked to/signposted from existing primary care templates; easy format to share with patients (QI‐ HCPs)	To allow rapid access to, and identification of, specific information and support required, with signposting to additional elements	Light touch ‘tailoring’ to direct towards most personally relevant content Very minimal amount of ‘tunnelled’ content Menu page style access with clear modules/sections Website optimized for use on mobile devices

Abbreviations: QI, qualitative interviews; SR, systematic review; ST, study team expert opinion; PPIE, public and patient involvement and engagement; YP, young people.

#### Intervention planning table including behavioural analysis

The intervention planning table is a further tool of the Person‐Based Approach which allows collation of theory and evidence obtained from the planning phase activities. This facilitates clear identification and documentation of key behaviours for the intervention to address, identified determinants of these behaviours, and intervention features and functions most likely to address these (Yardley et al., 2015).

The full intervention planning table and behavioural analysis is included in File S1. Acne Care Online targeted nine behaviours: initial sign up to the study, engagement with Acne Care Online digital content, consulting a health professional for advice and treatment, purchasing appropriate non‐prescribed treatments; instigating use of evidence‐based topical treatments, adherence to evidence‐based topical treatments, engaging in appropriate skincare behaviour alongside effective treatment use, utilizing appropriate psychological self‐management strategies, and seeking additional external emotional support. Mapping these behaviours, determinants, and proposed intervention features onto the BCW and TDF illustrates that Acne Care Online employs 23 different BCTs to deliver six intervention functions (modelling, education, persuasion, training, enablement, and environmental restructuring).

#### Intervention logic model

We used insights from the planning phase activities, and the resulting behavioural analysis and guiding principles, to construct the Acne Care Online logic model. The logic model represents the intervention's ‘programme theory’ which outlines how it is expected to achieve its intended outcomes, and the anticipated mechanisms through which this occurs (Funnell & Rogers, 2011). These hypotheses that the intervention should function through multiple components targeting a range of mechanisms including enhanced knowledge of acne and evidence‐based acne treatment options, more realistic outcome expectations, improved self‐efficacy for treatment seeking and use, reduced occurrence of side effects, and improved knowledge of strategies and support to minimize the mental health impact of acne. These features and mechanisms will be evaluated in subsequent process analyses to provide a more nuanced understanding of whether, and for whom, the intervention works and under what circumstances (Skivington et al., 2021).

**FIGURE 2:**
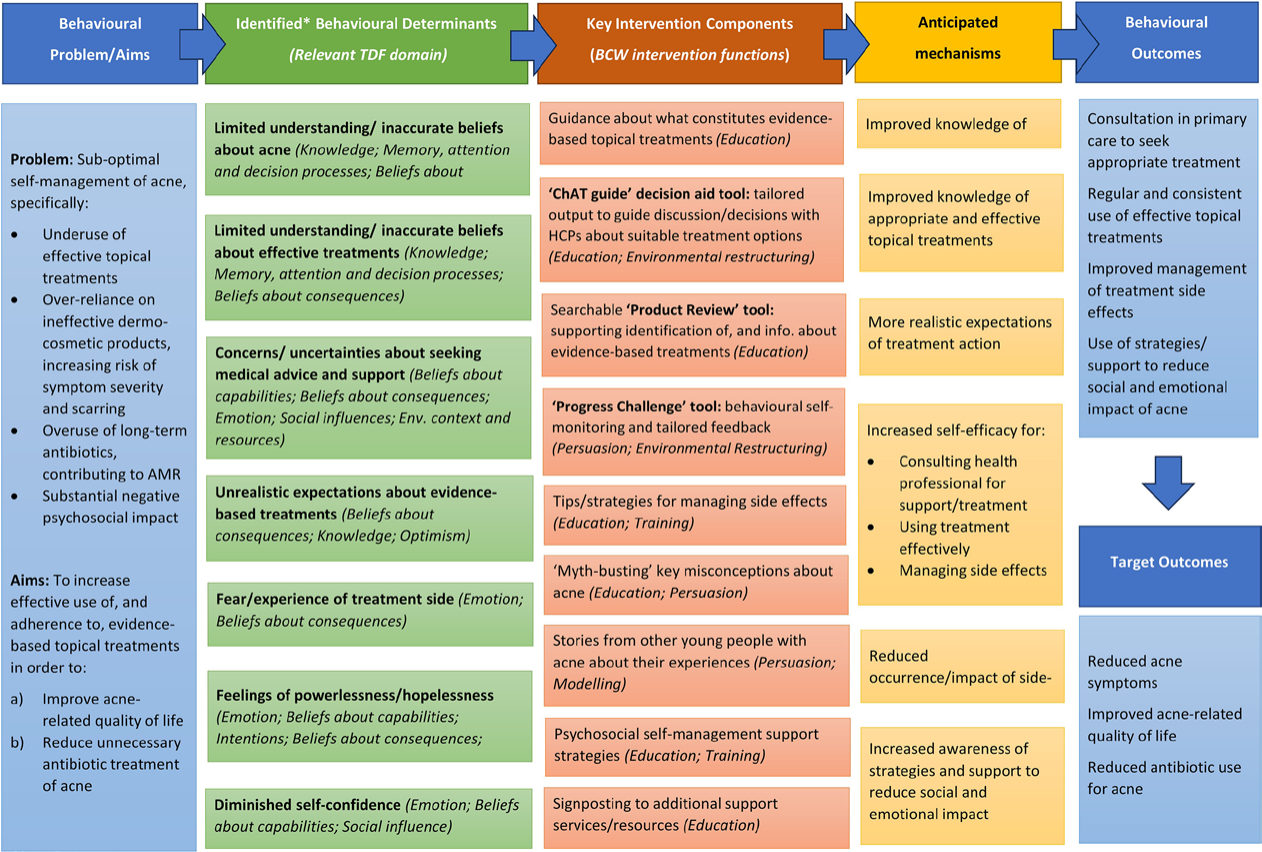
Logic model of the Acne Care Online intervention. BCW, Behaviour Change Wheel; HCPs, Healthcare Professionals; TDF, Theoretical Domains Framework; *Identified through intervention planning work including qualitative interviews, systematic reviews of literature, PPIE input and relevant theoretical models (Common Sense Model of Illness Perceptions; Necessity‐Concerns Framework; Social Cognitive Theory).

The Acne Care Online logic model is shown in Figure 2, below.

### Optimization

#### Participants

A diverse sample of 53 young people with current or recent experience of acne participated in a think‐aloud or longitudinal qualitative interview. Their characteristics are described in Table 2.

**TABLE 2:** Participant characteristics of optimization phase interviews.

Participant characteristic	Frequency
Gender	
Male	15
Female	37
Prefer not to say	1
Age	
13–15	16
16–18	17
19–21	9
22–25	11
Ethnicity	
White/White British	33
Asian/Asian British	14
Black/Black British	5
Not specified	1
IMD^a^	
1–5	17
6–10	33
Not specified	3
Region	
South West Central	15
South Central	14
North London	6
South London	4
East Midlands	4
West Midlands	3
North West	2
Yorkshire and Humber	1
East of England	1
South East	1
Not specified	2
Recruitment strategy	
Primary care	28
Social media	18
Resampled from planning phase	7
Duration of acne symptoms	
6 months to 1 year	7
Between 1 and 2 years	10
Over 2 years	36

Abbreviation: IMD, indices of multiple deprivation.

^a^ IMD: official measure of relative deprivation in England, expressed in deciles with top 10% (most deprived areas) being in the first decile (IMD = 1) and bottom 10% (least deprived areas) being in the tenth decile (IMD = 10).

#### Evidence of acceptability of acne care online content

In general, participants were very positive about the Acne Care Online content, reporting it to be comprehensive and relevant, providing novel, trustworthy information that was easy to understand.

Really helpful because it is so understandable. I went on it and I could picture myself on it as well, and picture myself actually using it, because there are loads of websites out there, but it's so confusing. […] so having it all in one place, step by step is so helpful. (TA022, Female, 18, Indian)

I think that it's very good. Lots of detail and there was loads of different methods that I didn't even know existed. So I think for someone that's really struggling, giving them this would really help them. (TA045, Male, 15, White British)

Several people appeared to particularly value the co‐produced nature of the content in ensuring the most salient issues were addressed:

People like me have been involved, so they understand, especially with the mental health and the effects on appearance and stuff like that, knowing that other young people have been involved in making it helps to know that those issues have been raised (TA056 Female, 19, White British)

There were also some early indications that individuals would be likely to implement changes to their behaviour in response to the content they had engaged with:

Yes, so maybe mini[mal]ising my skincare routine because it said on the website, you only really need cleanser… At one point it said three things that you need I think. It was cleanser, and then moisturiser was one of them. I just think it's quite interesting because on social media, skincare is quite romanticised to have a lot of skincare products and I think maybe that can be a bit harmful on your skin. So it's that nice to see it from a point of view from an actual GP or doctor website. (TA058, Female, 14, White British)

#### Issues identified and changes implemented

Throughout this iterative process, several minor changes were made to optimize the general usability, accessibility and clarity of intervention including reducing larger sections of text and adding additional navigational functionality. A smaller number of more substantial issues were identified, particularly around the communication of key messages, navigability of the intervention and definition of key terms. These are outlined in Table 3 alongside illustrative quotes and an explanation of how the issue was addressed. Following the implementation of these changes, further think‐aloud interviews indicated that these issues had been adequately addressed.

**TABLE 3:** Summary of key issues identified and actions taken to address these.

Summary of issue identified	Example	Change(s) implemented
Some uncertainty regarding how to navigate the intervention to find the most personally relevant content – sense of getting lost, or not knowing where to start	‘It feels like sometimes it's going round in circles, which… it is useful information that it's showing, coming up with, but maybe it can be a bit overwhelming having lots and lots of links’. (TA051, Female, 18, ‘Other’ ethnic group)	Personalization implemented in initial pages through brief tailoring questions to direct users to content likely to be most relevant to them. ‘Toolkit’ of key features, and main menu to access each core module made available from every page Addition of brief introductory ‘walkthrough’ video explaining how to navigate the website
Apparent that some key messages (e.g., that there is a difference between evidence‐based topical acne treatments and skincare products with no/insufficient active ingredients to treat acne) were not sufficiently clearly communicated and/or understood	‘I think it's clear in terms of skincare needs this and acne treatment needs this, but I would struggle to decide whether a product was a skincare product or an acne treatment product because I don't know from the page how to tell the difference between a product being one or the other.’ (TA001, Male, 16, White British) ‘Maybe make more pop‐ey boxes for key information…for me, if I saw that, unless it was highlighted in a bold font on keywords, I just would have browsed over that and brushed over it’ (TA052, Female, 20 White British)	Addition of ‘Important’ key message boxes highlighting core information and advice in brief text at the start of relevant sections
A number of people did not appear to understand key repeatedly used terminology – for example, ‘topical treatments’	‘I didn't realize there were different types of topical treatments. I thought there were just all the ones you put on your skin’ (TA009, Male, 22, Black Caribbean)	Where possible, we removed any specialist language and replaced this with a plain English alternative (e.g., ‘creams, gels or washes you put on your skin’). In instances where use of such terminology was unavoidable or would be overly wordy, these terms were made into clickable links that opened a ‘glossary’ pop‐up which defined the term
Some specific topics not sufficiently addressed (e.g., long‐term maintenance treatments and managing/avoiding scarring)	‘Also, I don't know if this is a useful thought or not, but I think sometimes when you stop using something, something can flare up again. So it can be helpful and then when you don't use it, it flares up again’ (TA030, Female, 24, Pakistani)	Addition of advice and guidance including: Direction about how/in what circumstance to continue long‐term use of topical treatments, including reassurance about safety of long‐term use Emphasizing the importance of early treatment for reducing risk of later scarring Information (including visual) about what scarring looks like on different skin types Advice about risk factors for scarring and how to avoid these

#### The acne care online intervention

The planning and optimization phases culminated in a version of the Acne Care Online digital intervention ready for evaluation of its feasibility and effectiveness. The intervention is described in the box below, with further detail provided in the TIDieR checklist (Hoffmann et al., 2014; File S2).

**Table:** 

The Acne Care Online Intervention
Acne Care Online is a standalone web application optimized for use on mobile devices. It comprises four core modules:
‘**Treatments that really work**’ focusing on supporting users to identify and access evidence‐based topical treatments, addressing common concerns and providing advice and support for avoiding and reducing side effects;
‘**Asking for help**’ provides advice to encourage and facilitate help‐seeking from the appropriate healthcare services, addressing common concerns, and how to make the most of consultations;
‘**Coping with acne**’ supporting users with managing the mental health impact of acne by providing guidance about self‐management strategies and signposting to additional support resources;

## DISCUSSION

This article provides an overview of the systematic theory‐, evidence‐, and person‐based approach to developing the Acne Care Online digital behaviour change intervention. It presents the methods and key findings of the qualitative interviews, systematic reviews, health behaviour change theory, and PPIE and stakeholder input which shaped the intervention's guiding principles, intervention planning table, and logic model. It also summarizes early user feedback that informed optimization of the draft materials, providing preliminary evidence that the Acne Care Online tool appears acceptable, relevant, and helpful for young people with acne.

Through the development processes, we identified important overarching issues for Acne Care Online to address to ensure that it is engaging, persuasive, and effective for supporting users to understand, access, and adhere to effective acne treatments. These were: (1) helping young people to understand the difference between evidence‐based acne treatments and generic skincare products; (2) providing encouragement and practical strategies for seeking help and advice; and (3) the need for a non‐judgemental approach aligned with encouragement to reduce physical and/or emotional discomfort, rather than motivations based on appearance concerns.

Insights from our initial qualitative interviews, public contributors, behaviour change theory and existing literature all indicated the importance of young people with acne fully understanding treatment options, and of having positive and realistic treatment expectations. However, the evidence collated also highlighted that in this context, there is often widespread uncertainty about what an acne treatment is, and that outcome expectations are frequently unrealistic. Our qualitative interview and PPIE activities supported previous suggestions that young people struggle to distinguish between evidence‐based acne treatments and dermo‐cosmetic style products (Araviiskaia et al., 2022; Ip et al., 2020) with many participants and contributors talking only about ‘off the shelf’ skincare products when asked how they manage their acne. Our findings provided further insight into how young people make decisions about which products to try, revealing that many rely on reviews from others – often online, but sometimes from people they know. This highlighted the potential for a searchable ‘product review’ feature which could be appealing in terms of providing ‘review’ type information, but also making a clear distinction between which products do and do not contain ingredients to actively treat acne, and setting clear expectations about how treatments work. The importance of such features to address this issue are supported by the common sense model of illness perceptions (CSM) (Diefenbach & Leventhal, 1996) and necessity concerns framework (NCF) (Horne, 1997, 2012; Horne & Weinman, 1999) which both indicate that an individual's beliefs about their treatments (including understanding how to use them, how important they think they are for treating acne, and their beliefs about whether they will work) are important determinants of their treatment‐related behaviours. These include actively seeking out the treatments, and the extent to which they adhere to their use.

Our development work also indicated the need for Acne Care Online to provide encouragement and practical support strategies for seeking medical help and advice for acne. Both young people themselves, and sometimes their parent/carer, expressed reservations about seeking professional medical attention for acne. This reflected findings from previous literature (Corey et al., 2013; Desai et al., 2017; Ip et al., 2020; McNiven, 2018) and was largely due to perceptions that acne was not serious enough, or uncertainty about how to approach a consultation. This indicated the need for intervention features to enhance young people's confidence in their ability to seek support and which address concerns and misconceptions about acne not warranting medical attention. Self‐efficacy theory suggests that verbal persuasion and vicarious experience (observing similar others modelling the desired behaviour) are useful techniques for enhancing self‐efficacy for performing a given behaviour (Bandura, 1977). Acne Care Online draws on these techniques with features such as video clips from health professionals explaining the importance of consulting early about acne (verbal persuasion). It also contains stories from other young people with acne about their positive experiences of consulting (vicarious experience). In addition to features to support and encourage medical help‐seeking, Acne Care Online features a user‐generated personalized acne treatment decision‐aid tool to facilitate communication of an individual's specific needs and preferences during a consultation. This aligns with recent recommendations for more collaborative and holistic approaches to acne management in which patients are involved in decision‐making (Layton et al., 2023). Evidence from a Cochrane review indicates that individuals with access to personalized decision aids feel more knowledgeable and informed about their condition and have a more active role in treatment decision‐making (Stacey et al., 2017).

Finally, the development process revealed the importance of Acne Care Online adopting a non‐judgemental approach, emphasizing that individuals should make an informed decision to address their acne based on reducing distress or discomfort, rather than conforming to appearance‐related expectations. This was most closely informed by our qualitative interviews and input from public contributors, which revealed that, despite the majority of interviewees recognizing the detrimental impact of acne on their mental health, many were also increasingly aware of, and engaged with, a more ‘body/skin positive or neutral’ outlook. This often developed over time with experience of living with acne, but sometimes arose initially from engagement with social media platforms. In line with recent work seeking to define the concept of ‘body neutrality’ (Pellizzer & Wade, 2023), this perspective appeared to promote a sense of acceptance and understanding that appearance does not dictate a person's value or worth. These insights align with the popularity of ‘acne positivity’ social media content (Iyengar et al., 2025), highlighting the need to be sensitive in approaching communication about the importance of treating and managing acne. This informed the development of the Acne Care Online module which supports the management of the mental health impact of acne, by offering self‐management strategies and techniques aiming to empower the individual. This included techniques for building confidence in themselves and amongst other people, practising self‐compassion, and managing stress and challenging social situations, supplemented by signposting to additional support. Many of these approaches are recognized as promising strategies for managing the mental health impact of acne (Hughes & Bewley, 2023).

Our findings provide important preliminary evidence that our digital behaviour change intervention for acne is acceptable and accessible for intended users, with early indications from interviews with HCPs that implementation within current practice should be possible. Furthermore, it indicates that the core messages and features developed appear relevant, persuasive, and informative amongst young people with acne. These findings also have important implications for how clinicians engage with young people with acne – in particular, the need to reassure young people that seeking help for acne is appropriate, and that their expectations and understandings of acne treatments are accurate and realistic. The work also highlights the value of using relevant behaviour change theory to inform interventions for supporting help‐seeking and adherence‐related behaviours in the context of self‐managing health conditions.

The strengths of this work lie in the systematic theory‐, evidence‐, and person‐based development approach. Such rigorous approaches make the development process transparent and subsequently enable more informed process evaluation to allow a fuller understanding of the intervention's mechanisms (O'Cathain et al., 2019; Skivington et al., 2021). They also permit triangulation between different sources of evidence to maximize confidence in our findings (Rigour, 2023). Furthermore, the depth and breadth of the PPIE input, including a diverse range of young people, maximizes the chances that Acne Care Online will be relevant, engaging, and useful for young people from different backgrounds and life circumstances. This also facilitated member checking of data and interpretations at all stages of the development process, further contributing to the credibility of findings (Rigour, 2023). Despite best attempts, recruiting boys and young men with acne remained challenging. It is unclear whether this is due to these individuals feeling less affected by their acne, and so perhaps seeing such a study as less relevant. Preliminary evidence from a recent European study indicates that females report significantly higher detrimental impact of acne on quality of life, stigmatization and anxiety relative to male counterparts, even when their acne is perceived to be less severe (Szepietowska et al., 2024). However, consideration should also be given to whether boys and young men feel less willing and/or able to discuss and share their experiences on the topic, or to take part in research. In the present study, all team members involved in recruiting and interviewing participants were female which may have had further bearing on whether boys and young men felt inclined to participate. Further work investigating boys and young men's experiences of acne may benefit from exploring novel means of recruiting and collecting data that may be more appealing amongst this group.

## CONCLUSIONS

More self‐management support is required for young people with acne to understand, access, and effectively use evidence‐based topical treatments. We have outlined the planning and optimization processes of the Acne Care Online digital behaviour change intervention to address this need. As well as providing a methodological model for informing intervention development, this work provides important evidence of the specific intervention's acceptability and relevance for target users. The findings also provide important insights for clinicians managing acne. The work described in this process illustrates how behaviour change theory can be effectively applied to developing interventions to support medical help‐seeking and treatment adherence. Further work will evaluate the feasibility, effectiveness, and cost‐effectiveness of Acne Care Online for improving young people's acne severity and quality of life.

## AUTHOR CONTRIBUTIONS

**Rosie Essery:** Data curation; formal analysis; investigation; methodology; project administration; resources; supervision; visualization; writing – review and editing; writing – original draft. **Mary Steele:** Formal analysis; investigation; methodology; resources; software; supervision; writing – review and editing. **Stephanie Easton:** Formal analysis; investigation; methodology; resources; writing – review and editing. **Sebastien Pollet:** Formal analysis; resources; software; writing – review and editing. **Charlotte Cairns:** Investigation; writing – review and editing. **Rebekah LeFeuvre:** Project administration; data curation; writing – review and editing. **Julie Hooper:** Project administration; writing – review and editing. **Taeko Becque:** Formal analysis; investigation; writing – review and editing. **Tanith Kane:** Formal analysis; writing – review and editing; investigation. **Georgina Hart:** Investigation; formal analysis; writing – review and editing. **Tracey Sach:** Conceptualization; methodology; funding acquisition; writing – review and editing; resources. **Nick Francis:** Conceptualization; funding acquisition; methodology; writing – review and editing. **Paul Little:** Conceptualization; funding acquisition; methodology; writing – review and editing. **Lucy Yardley:** Conceptualization; methodology; writing – review and editing; funding acquisition. **Sophie Dove:** Conceptualization; writing – review and editing; funding acquisition; resources. **Kate Henaghan‐Sykes:** Resources; writing – review and editing. **Irene Soulsby:** Resources; writing – review and editing; conceptualization; funding acquisition. **Kim S. Thomas:** Conceptualization; funding acquisition; methodology; writing – review and editing. **Matthew J. Ridd:** Conceptualization; methodology; funding acquisition; writing – review and editing. **Beth Stuart:** Conceptualization; methodology; funding acquisition; writing – review and editing. **Alison M. Layton:** Conceptualization; methodology; funding acquisition; writing – review and editing. **Andrew R. Thompson:** Conceptualization; methodology; funding acquisition; resources; writing – review and editing. **Mahendra Patel:** Conceptualization; methodology; writing – review and editing; funding acquisition. **Adam Yates:** Resources; writing – review and editing. **Miriam Santer:** Conceptualization; methodology; formal analysis; funding acquisition; project administration; supervision; resources; writing – review and editing. **Ingrid Muller:** Conceptualization; methodology; writing – review and editing; funding acquisition; project administration; supervision; resources; formal analysis.

## Supporting information


File S1.

File S2.

## Data Availability

The data that support the findings of this study are available from the corresponding author upon reasonable request.

## References

[bjhp70033-bib-0001] Alexis, A. F. , Woolery‐Lloyd, H. , Williams, K. , Andriessen, A. , Callender, V. D. , Kang, S. , Rodriquez, D. , & Tan, J. (2021). Racial/ethnic variations in acne: Implications for treatment and skin care recommendations for acne patients with skin of color. Journal of Drugs in Dermatology, 20(7), 716–725. 34232006 10.36849/JDD.6169

[bjhp70033-bib-0002] Araviiskaia, E. , Layton, A. M. , Estebaranz, J. L. L. , Ochsendorf, F. , & Micali, G. (2022). The synergy between pharmacological regimens and Dermocosmetics and its impact on adherence in acne treatment. Dermatology Research and Practice, 2022(1), 3644720. 35982914 10.1155/2022/3644720 PMC9381271

[bjhp70033-bib-0003] Bandura, A. (1977). Self‐efficacy: toward a unifying theory of behavioral change. Psychological Review, 84(2), 191. 847061 10.1037//0033-295x.84.2.191

[bjhp70033-bib-0004] Barlow, R. , Payyazhi, G. , Hogan, S. , Grindlay, D. , Choi, D. , Verma, M. , Pasunuru, K. , Taylor, R. , Bewley, A. , & Mohandas, P. (2023). Suicide and suicidality in children and adolescents with chronic skin disorders: A systematic review. Acta Dermato‐Venereologica, 103, adv00851. 36629476 10.2340/actadv.v102.1502 PMC9885289

[bjhp70033-bib-0005] Bhate, K. , Mansfield, K. E. , Sinnott, S.‐J. , Margolis, D. J. , Adesanya, E. , Francis, N. , Leyrat, C. , Hopkins, S. , Stabler, R. , Shallcross, L. , Langan, S. M. , & Mathur, R. (2022). Long‐term oral antibiotic use in people with acne vulgaris in UK primary care: a drug utilization study. British Journal of Dermatology, 188(3), 361–371. 10.1093/bjd/ljac084 36670540

[bjhp70033-bib-0006] Bienenfeld, A. , Nagler, A. R. , & Orlow, S. J. (2017). Oral antibacterial therapy for acne vulgaris: An evidence‐based review. American Journal of Clinical Dermatology, 18(4), 469–490. 28255924 10.1007/s40257-017-0267-z

[bjhp70033-bib-0007] Bradbury, K. , Morton, K. , Band, R. , van Woezik, A. , Grist, R. , McManus, R. J. , Little, P. , & Yardley, L. (2018). Using the person‐based approach to optimise a digital intervention for the management of hypertension. PLoS One, 13(5), e0196868. 29723262 10.1371/journal.pone.0196868 PMC5933761

[bjhp70033-bib-0008] Braun, V. , & Clarke, V. (2006). Using thematic analysis in psychology. Qualitative Research in Psychology, 3(2), 77–101.

[bjhp70033-bib-0009] Cane, J. , O'Connor, D. , & Michie, S. (2012). Validation of the theoretical domains framework for use in behaviour change and implementation research. Implementation Science, 7, 37. 22530986 10.1186/1748-5908-7-37 PMC3483008

[bjhp70033-bib-0010] Corcoran, L. , Muller, I. , Layton, A. M. , Rucinski, G. , Venkatess, V. , Sufraz, A. , Dove, S. , Lown, M. , Stuart, B. , Francis, N. , & Santer, M. (2023). Systematic review of clinical practice guidelines for acne vulgaris published between January 2017 and July 2021. Skin Health and Disease, 3(4), e240. 37538340 10.1002/ski2.240 PMC10395621

[bjhp70033-bib-0011] Corey, K. C. , Cheng, C. E. , Irwin, B. , & Kimball, A. B. (2013). Self‐reported help‐seeking behaviors and treatment choices of adolescents regarding acne. Pediatric Dermatology, 30(1), 36–41. 22888857 10.1111/j.1525-1470.2012.01807.x

[bjhp70033-bib-0012] Desai, K. P. , Martyn‐Simmons, C. , Viner, R. , & Segal, T. (2017). Help‐seeking behaviours, opportunistic treatment and psychological implications of adolescent acne: cross‐sectional studies in schools and hospital outpatient departments in the UK. BMJ Open, 7(9), e016964. 10.1136/bmjopen-2017-016964 PMC5623513 28939579

[bjhp70033-bib-0013] Diefenbach, M. A. , & Leventhal, H. (1996). The common‐sense model of illness representation: Theoretical and practical considerations. Journal of Social Distress and Homelessness, 5(1), 11–38.

[bjhp70033-bib-0014] Donnarumma, M. , Fattore, D. , Greco, V. , Ferrillo, M. , Vastarella, M. , Chiodini, P. , & Fabbrocini, G. (2019). How to increase adherence and compliance in acne treatment? A combined strategy of SMS and visual instruction leaflet. Dermatology, 235(6), 463–470. 31586999 10.1159/000502575

[bjhp70033-bib-0015] Dréno, B. , Thiboutot, D. , Gollnick, H. , Finlay, A. Y. , Layton, A. , Leyden, J. J. , Leutenegger, E. , Perez, M. , & Global Alliance to Improve Outcomes in Acne . (2010). Large‐scale worldwide observational study of adherence with acne therapy. International Journal of Dermatology, 49(4), 448–456. 20465705 10.1111/j.1365-4632.2010.04416.x

[bjhp70033-bib-0016] Eichenfield, D. Z. , Sprague, J. , & Eichenfield, L. F. (2021). Management of Acne Vulgaris: A review. Journal of the American Medical Association, 326(20), 2055–2067. 34812859 10.1001/jama.2021.17633

[bjhp70033-bib-0017] Ewulu, A. R. , Singh, R. , Roberson, K. B. , Masicampo, E. J. , & Feldman, S. R. (2024). Toward a better understanding of treatment adherence: incorporating accountability explicitly into the social cognitive theory of adherence behavior. Journal of Dermatological Treatment, 35(1), 2351493. 38719206 10.1080/09546634.2024.2351493

[bjhp70033-bib-0018] Francis, N. A. , Entwistle, K. , Santer, M. , Layton, A. M. , Eady, E. A. , & Butler, C. C. (2017). The management of acne vulgaris in primary care: a cohort study of consulting and prescribing patterns using the clinical practice research datalink. British Journal of Dermatology, 176(1), 107–115. 27716910 10.1111/bjd.15081

[bjhp70033-bib-0019] Funnell, S. C. , & Rogers, P. J. (2011). Purposeful program theory: Effective use of theories of change and logic models. John Wiley and Sons.

[bjhp70033-bib-0020] Goff, G. K. , & Stein, S. L. (2025). Cosmeceuticals in the pediatric population part I: A review of risks and available evidence. Pediatric Dermatology, 42(2), 221–227. 39925031 10.1111/pde.15866 PMC11950811

[bjhp70033-bib-0021] Gollnick, H. P. , Bettoli, V. , Lambert, J. , Araviiskaia, E. , Binic, I. , Dessinioti, C. , Galadari, I. , Ganceviciene, R. , Ilter, N. , Kaegi, M. , Kemeny, L. , López‐Estebaranz, J. L. , Massa, A. , Oprica, C. , Sinclair, W. , Szepietowski, J. C. , & Dréno, B. (2016). A consensus‐based practical and daily guide for the treatment of acne patients. Journal of the European Academy of Dermatology and Venereology, 30(9), 1480–1490. 27177989 10.1111/jdv.13675

[bjhp70033-bib-0022] Greenwell, K. , Becque, T. , Sivyer, K. , Steele, M. , Denison‐Day, J. , Howells, L. , Ridd, M. J. , Roberts, A. , Lawton, S. , Langan, S. M. , Hooper, J. , Wilczynska, S. , Griffiths, G. , Sach, T. H. , Little, P. , Williams, H. C. , Thomas, K. S. , Yardley, L. , Muller, I. , … Stuart, B. (2024). Online behavioural interventions for children and young people with eczema: a quantitative evaluation. British Journal of General Practice, 74(743), e379–e386. 10.3399/BJGP.2023.0411 PMC11104514 38316467

[bjhp70033-bib-0023] Greenwell, K. , Sivyer, K. , Howells, L. , Steele, M. , Ridd, M. J. , Roberts, A. , Ahmed, A. , Lawton, S. , Langan, S. M. , Hooper, J. , Wilczynska, S. , Leighton, P. , Griffiths, G. , Sach, T. , Little, P. , Williams, H. C. , Thomas, K. S. , Yardley, L. , Santer, M. , & Muller, I. (2022). ‘Eczema shouldn't control you; you should control eczema’: qualitative process evaluation of online behavioural interventions to support young people and parents/carers of children with eczema. British Journal of Dermatology, 188(4), 506–513. 10.1093/bjd/ljac115 36745562

[bjhp70033-bib-0024] Hagger, M. S. , & Orbell, S. (2003). A meta‐analytic review of the common‐sense model of illness representations. Psychology and Health, 18(2), 141–184.

[bjhp70033-bib-0025] Heng, A. H. S. , & Chew, F. T. (2020). Systematic review of the epidemiology of acne vulgaris. Scientific Reports, 10(1), 5754. 32238884 10.1038/s41598-020-62715-3 PMC7113252

[bjhp70033-bib-0026] Hoffmann, T. C. , Glasziou, P. P. , Boutron, I. , Milne, R. , Perera, R. , Moher, D. , Altman, D. G. , Barbour, V. , Macdonald, H. , Johnston, M. , Lamb, S. E. , Dixon‐Woods, M. , McCulloch, P. , Wyatt, J. C. , Chan, A. W. , & Michie, S. (2014). Better reporting of interventions: template for intervention description and replication (TIDieR) checklist and guide. BMJ, 348, g1687. 24609605 10.1136/bmj.g1687

[bjhp70033-bib-0027] Holden, G. (1992). The relationship of self‐efficacy appraisals to subsequent health related outcomes. Social Work in Health Care, 16(1), 53–93. 1839087 10.1300/j010v16n01_05

[bjhp70033-bib-0028] Horne, R. (1997). Representations of medication and treatment: advances in theory and measurement. In K. J. Petrie & J. A. Weinman (Eds.), Perceptions of Health and Illness (pp. 155–188). Harwood Academic Publishers.

[bjhp70033-bib-0029] Horne, R. (2012). Treatment perceptions and self‐regulation. In The self‐regulation of health and illness behaviour (pp. 138–154). Routledge.

[bjhp70033-bib-0030] Horne, R. , Chapman, S. C. , Parham, R. , Freemantle, N. , Forbes, A. , & Cooper, V. (2013). Understanding patients’ adherence‐related beliefs about medicines prescribed for long‐term conditions: a meta‐analytic review of the necessity‐concerns framework. PLoS One, 8(12), e80633. 24312488 10.1371/journal.pone.0080633 PMC3846635

[bjhp70033-bib-0031] Horne, R. , & Weinman, J. (1999). Patients' beliefs about prescribed medicines and their role in adherence to treatment in chronic physical illness. Journal of Psychosomatic Research, 47(6), 555–567. 10661603 10.1016/s0022-3999(99)00057-4

[bjhp70033-bib-0032] Hughes, O. , & Bewley, A. (2023). Is it really ever ‘just acne’? Considering the psychodermatology of acne. British Journal of Dermatology, 189(Supplement_1), i11–i16. 37903071 10.1093/bjd/ljad251

[bjhp70033-bib-0033] Ip, A. , Muller, I. , Geraghty, A. W. A. , McNiven, A. , Little, P. , & Santer, M. (2020). Young people's perceptions of acne and acne treatments: secondary analysis of qualitative interview data. The British Journal of Dermatology, 183(2), 349–356. 31701523 10.1111/bjd.18684 PMC7496424

[bjhp70033-bib-0034] Ip, A. , Muller, I. , Geraghty, A. W. A. , Platt, D. , Little, P. , & Santer, M. (2021). Views and experiences of people with acne vulgaris and healthcare professionals about treatments: systematic review and thematic synthesis of qualitative research. BMJ Open, 11(2), e041794. 10.1136/bmjopen-2020-041794 PMC7853035 33526498

[bjhp70033-bib-0035] Ip, A. , Muller, I. , Geraghty, A. W. A. , Rumsby, K. , Stuart, B. , Little, P. , & Santer, M. (2021). Supporting self‐management among young people with acne vulgaris through a web‐based behavioral intervention: Development and feasibility randomized controlled trial. JMIR Dermatology, 4(2), e25918. 37632804 10.2196/25918 PMC10334953

[bjhp70033-bib-0036] Iyengar, L. , Saldanha, S. , & Chong, A. H. (2025). #Acne: A thematic qualitative analysis of acne content on TikTok. Australasian Journal of Dermatology, 66(3), 127–134. 40008491 10.1111/ajd.14433 PMC12062723

[bjhp70033-bib-0037] Kok, G. , & Schaalma, H. (2004). Using theory in psychological interventions. In S. Michie & C. Abraham (Eds.), Health psychology in practice (pp. 201–229). Blackwell Publishing.

[bjhp70033-bib-0038] Layton, A. M. , Alexis, A. , Baldwin, H. , Bettoli, V. , Del Rosso, J. , Dirschka, T. , Dréno, B. , Gold, L. S. , Harper, J. , Ko, J. Y. , Al Nuaimi, K. , Oon, H. H. , Rajagopalan, M. , Rocha, M. , See, J. A. , Weiss, J. , & Tan, J. (2023). The personalized acne treatment tool—Recommendations to facilitate a patient‐centered approach to acne management from the personalizing acne: Consensus of experts. JAAD International, 12, 60–69. 37274381 10.1016/j.jdin.2023.03.013 PMC10236180

[bjhp70033-bib-0039] Layton, A. M. , Thiboutot, D. , & Tan, J. (2021). Reviewing the global burden of acne: how could we improve care to reduce the burden?*. British Journal of Dermatology, 184(2), 219–225. 32770673 10.1111/bjd.19477

[bjhp70033-bib-0040] Leung, A. K. , Barankin, B. , Lam, J. M. , Leong, K. F. , & Hon, K. L. (2021). Dermatology: how to manage acne vulgaris. Drugs Context, 10, 2021‐8‐6. 10.7573/dic.2021-8-6 PMC8510514 34691199

[bjhp70033-bib-0041] Marasca, C. , Ruggiero, A. , Fontanella, G. , Ferrillo, M. , Fabbrocini, G. , & Villani, A. (2020). Telemedicine and support groups could be used to improve adherence to treatment and health‐related quality of life in patients affected by inflammatory skin conditions during the COVID‐19 pandemic. Clinical and Experimental Dermatology, 45(6), 749. 10.1111/ced.14245 PMC7264494 32304587

[bjhp70033-bib-0042] McNiven, A. (2018). ‘Disease, illness, affliction? Don't know’: Ambivalence and ambiguity in the narratives of young people about having acne. Health, 23(3), 273–288. 29552892 10.1177/1363459318762035

[bjhp70033-bib-0043] Michie, S. , Richardson, M. , Johnston, M. , Abraham, C. , Francis, J. , Hardeman, W. , Eccles, M. P. , Cane, J. , & Wood, C. E. (2013). The behavior change technique taxonomy (v1) of 93 hierarchically clustered techniques: Building an international consensus for the reporting of behavior change interventions. Annals of Behavioral Medicine, 46(1), 81–95. 23512568 10.1007/s12160-013-9486-6

[bjhp70033-bib-0044] Michie, S. , Van Stralen, M. M. , & West, R. (2011). The behaviour change wheel: a new method for characterising and designing behaviour change interventions. Implementation Science, 6, 42. 21513547 10.1186/1748-5908-6-42 PMC3096582

[bjhp70033-bib-0045] Michie, S. , Yardley, L. , West, R. , Patrick, K. , & Greaves, F. (2017). Developing and evaluating digital interventions to promote behavior change in health and health care: Recommendations resulting from an international workshop. Journal of Medical Internet Research, 19(6), e232. 28663162 10.2196/jmir.7126 PMC5509948

[bjhp70033-bib-0046] Muller, I. , Santer, M. , Morrison, L. , Morton, K. , Roberts, A. , Rice, C. , Williams, M. , & Yardley, L. (2019). Combining qualitative research with PPI: reflections on using the person‐based approach for developing behavioural interventions. Research Involvement and Engagement, 5(1), 34. 31807316 10.1186/s40900-019-0169-8 PMC6857167

[bjhp70033-bib-0047] National Health Service . (2025). Acne Treatment (NHS webpages). https://www.nhs.uk/conditions/acne/treatment/

[bjhp70033-bib-0049] O'Cathain, A. , Croot, L. , Sworn, K. , Duncan, E. , Rousseau, N. , Turner, K. , Yardley, L. , & Hoddinott, P. (2019). Taxonomy of approaches to developing interventions to improve health: a systematic methods overview. Pilot and Feasibility Studies, 5(1), 41. 30923626 10.1186/s40814-019-0425-6 PMC6419435

[bjhp70033-bib-0050] Office for National Statistics . (2021). Internet Users, UK: 2020.

[bjhp70033-bib-0052] Park, E. , & Kwon, M. (2018). Health‐related internet use by children and adolescents: Systematic review. Journal of Medical Internet Research, 20(4), e120. 29615385 10.2196/jmir.7731 PMC5904452

[bjhp70033-bib-0053] Pellizzer, M. L. , & Wade, T. D. (2023). Developing a definition of body neutrality and strategies for an intervention. Body Image, 46, 434–442. 37573765 10.1016/j.bodyim.2023.07.006

[bjhp70033-bib-0054] Rigour, A. D. (2023). Qualitative research–A practical guide for health and social care researchers and practitioners. Council of Australian University Librarians, Open Educational Resources Collective.

[bjhp70033-bib-0055] Russell, J. , Greenhalgh, T. , & Taylor, M. (2019). Patient and public involvement in NIHR research 2006–2019: policy intentions, progress and themes. National Institute for Health Research.

[bjhp70033-bib-0056] Sach, T. H. , Onoja, M. , Clarke, H. , Santer, M. , Muller, I. , Becque, T. , Stuart, B. , Hooper, J. , Steele, M. , Wilczynska, S. , Ridd, M. J. , Roberts, A. , Ahmed, A. , Yardley, L. , Little, P. , Greenwell, K. , Sivyer, K. , Nuttall, J. , Griffiths, G. , … Thomas, K. S. (2024). Cost‐effectiveness of two online interventions supporting self‐care for eczema for parents/carers and young people. The European Journal of Health Economics, 25, 1165–1176. 38194207 10.1007/s10198-023-01649-9 PMC11377600

[bjhp70033-bib-0057] Samuels, D. V. , Rosenthal, R. , Lin, R. , Chaudhari, S. , & Natsuaki, M. N. (2020). Acne vulgaris and risk of depression and anxiety: A meta‐analytic review. Journal of the American Academy of Dermatology, 83(2), 532–541. 32088269 10.1016/j.jaad.2020.02.040

[bjhp70033-bib-0058] Santer, M. , Muller, I. , Becque, T. , Stuart, B. , Hooper, J. , Steele, M. , Wilczynska, S. , Sach, T. H. , Ridd, M. J. , Roberts, A. , Ahmed, A. , Yardley, L. , Little, P. , Greenwell, K. , Sivyer, K. , Nuttall, J. , Griffiths, G. , Lawton, S. , Langan, S. M. , … Thomas, K. S. (2022). Eczema care online behavioural interventions to support self‐care for children and young people: two independent, pragmatic, randomised controlled trials. BMJ, 379, e072007. 36740888 10.1136/bmj-2022-072007 PMC11778922

[bjhp70033-bib-0059] Skivington, K. , Matthews, L. , Simpson, S. A. , Craig, P. , Baird, J. , Blazeby, J. M. , Boyd, K. A. , Craig, N. , French, D. P. , McIntosh, E. , Petticrew, M. , Rycroft‐Malone, J. , White, M. , & Moore, L. (2021). A new framework for developing and evaluating complex interventions: update of Medical Research Council guidance. BMJ, 374, n2061. 34593508 10.1136/bmj.n2061 PMC8482308

[bjhp70033-bib-0060] Stacey, D. , Légaré, F. , Lewis, K. , Barry, M. J. , Bennett, C. L. , Eden, K. B. , Holmes‐Rovner, M. , Llewellyn‐Thomas, H. , Lyddiatt, A. , Thomson, R. , & Trevena, L. (2017). Decision aids for people facing health treatment or screening decisions. Cochrane Database of Systematic Reviews, 4, CD001431. 28402085 10.1002/14651858.CD001431.pub5 PMC6478132

[bjhp70033-bib-0061] Stuart, B. , Maund, E. , Wilcox, C. , Sridharan, K. , Sivaramakrishnan, G. , Regas, C. , Newell, D. , Soulsby, I. , Tang, K. F. , Finlay, A. Y. , Bucher, H. C. , Little, P. , Layton, A. M. , & Santer, M. (2021). Topical preparations for the treatment of mild‐to‐moderate acne vulgaris: systematic review and network meta‐analysis*. British Journal of Dermatology, 185(3), 512–525. 33825196 10.1111/bjd.20080

[bjhp70033-bib-0062] Szepietowska, M. , Stefaniak, A. A. , Krajewski, P. K. , & Matusiak, L. (2024). Females may have less severe acne, but they suffer more: A prospective cross‐sectional study on psychosocial consequences in 104 consecutive polish acne patients. Journal of Clinical Medicine, 13(1), 4. 10.3390/jcm13010004 PMC10779808 38202011

[bjhp70033-bib-0063] Tan, J. , Beissert, S. , Cook‐Bolden, F. , Chavda, R. , Harper, J. , Hebert, A. , Lain, E. , Layton, A. , Rocha, M. , Weiss, J. , & Dréno, B. (2021). Impact of facial and truncal acne on quality of life: A multi‐country population‐based survey. JAAD International, 3, 102–110. 34409378 10.1016/j.jdin.2021.03.002 PMC8362284

[bjhp70033-bib-0064] Thiboutot, D. , Dréno, B. , & Layton, A. (2008). Acne counseling to improve adherence. Cutis, 81(1), 81–86. 18306854

[bjhp70033-bib-0065] Van den Haak, M. J. , De Jong, M. D. , & Schellens, P. J. (2007). Evaluation of an informational web site: three variants of the think‐aloud method compared. Technical Communication, 54(1), 58–71.

[bjhp70033-bib-0066] Walsh, T. R. , Efthimiou, J. , & Dréno, B. (2016). Systematic review of antibiotic resistance in acne: an increasing topical and oral threat. The Lancet Infectious Diseases, 16(3), e23–e33. 26852728 10.1016/S1473-3099(15)00527-7

[bjhp70033-bib-0067] Wang, A. S. , Wu, J. , Tuong, W. , Schupp, C. , & Armstrong, A. W. (2015). Effectiveness of a novel interactive health care education tool on clinical outcomes and quality of life in acne patients: A randomized controlled pilot study. Journal of Dermatological Treatment, 26(5), 435–439. 25790848 10.3109/09546634.2015.1020915

[bjhp70033-bib-0068] Wilcock, J. , Kuznetsov, L. , Ravenscroft, J. , Rafiq, M. I. , & Healy, E. (2021). New NICE guidance on acne vulgaris: implications for first‐line management in primary care. British Journal of General Practice, 71(713), 568–570. 10.3399/bjgp21X717977 PMC8686441 34824079

[bjhp70033-bib-0069] Williams, H. C. , Dellavalle, R. P. , & Garner, S. (2012). Acne vulgaris. The Lancet, 379(9813), 361–372. 10.1016/S0140-6736(11)60321-8 21880356

[bjhp70033-bib-0070] Yardley, L. , Morrison, L. , Bradbury, K. , & Muller, I. (2015). The person‐based approach to intervention development: Application to digital health‐related behavior change interventions. Journal of Medical Internet Research, 17(1), e30. 25639757 10.2196/jmir.4055 PMC4327440

