# Supplementary material for: Planning and optimizing a digital self‐management support intervention: Acne Care Online
Source: Br J Health Psychol. 2025 Nov 5;30(4):e70033. doi: 10.1111/bjhp.70033 (PMC12587102; doi:10.1111/bjhp.70033)
Supplement: Supplementary file 1 — File S1. [file BJHP-30-0-s001.docx]

**Supplementary File 1.** Behavioural analysis of Acne Care Online (ACO)

| **Barrier/ *facilitator* to target behaviour**  **(source of evidence*)** | **Intervention/study component** | **Target construct (BCW)** | **Key TDF domains**  **relevant** | **Intervention function (BCW)** | **BCT (using 93 BCTs on BCT taxonomy v1)** |
| --- | --- | --- | --- | --- | --- |
| **Behaviour: Signing up to Acne Care Online study** | | | | | |
| Low health and/or digital literacy **(ST)**  Limited access to internet enabled-device (**ST)** | Recruitment materials and study procedures use clear simple language and navigation to ensure readability and accessibility (iterated with PPI and users)  Potential benefits of study clearly explained in accessible language  Brief video explaining website layout and navigation | Psychological capability  Physical opportunity | Knowledge; Cognitive and interpersonal skills  Environmental context and resources | Education; Training; Environmental restructuring | 4.1. Instruction on how to perform the behaviour  5.1. Information about health consequences |
| Privacy and credibility concerns **(ST; PPIE)** | Direct contact details (email/phone/SMS) for study team provided for questions and further information  University and NHS logos and approval reference details provided on recruitment materials and website pages  Only essential (for study purposes) information collected and stored and clear statement provided about how data is collected, stored and used | Reflective motivation  Psychological capability | Beliefs about consequences  Knowledge; Memory, attention and decision processes | Education; Persuasion | 5.1. Information about health consequences  9.1 Credible source  15.2 Social support (practical) |
| Limited attention span/preference for minimal text **(PPIE; QI)**  *Interest in study* **(PPIE; QI)**  *Acne symptoms impacting mental health and wellbeing* **(PPIE; QI)**  *Encouragement from parent/family member* **(PPIE; QI)**  *Altruism* **(PPIE; QI)** | Persuasive recruitment materials highlighting benefits of study  Brief recruitment materials with minimal text  More information accessible via QR code/direct URL for quick/easy access  Direct contact details (email/phone/SMS) for study team provided for questions and further information  Mobile optimised website (study processes and intervention) to allow study completion on mobile | Psychological capability  Reflective motivation  Automatic motivation  Social opportunity | Memory, attention and decision processes  Intentions; Goals; Beliefs about consequences  Emotion  Social influences | Environmental restructuring; Education; Persuasion | 5.1. Information about health consequences  15.2 Social support (practical) |
| **Engagement with Acne Care Online digital content over duration of study** | | | | | |
| Low health and/or digital literacy **(ST)**  *Perceived utility/convenience of online technology* (**PPIE; QI)** | Simple login procedures with instructions (iterated with PPI and users)  Easily navigable website (iterated with PPI and users)  Weekly SMS/email prompts to return to ACO (including URL) advertising different elements of ACO that may not have been viewed  Brief video explaining website layout and navigation | Psychological capability  Physical opportunity | Knowledge; Cognitive and interpersonal skills  Environmental context and resources | Education; Training; Environmental restructuring | 4.1. Instruction on how to perform the behaviour  7.1 Prompts/cues |
| Competing priorities/limited time **(QI; PPIE)**  Forgetting **(QI; PPIE)** | Automated reminders if user has not logged in after registration  Weekly SMS/email prompts to return to ACO (including URL) advertising different elements of ACO that may not have been viewed | Physical opportunity  Psychological capability | Environmental context and resources  Memory, attention and decision processes | Environmental restructuring | 7.1 Prompts/cues |
| Perception of no new information/ not learning anything new **(PPIE;QI)**  Can’t find advice on a specific topic of interest **(QI: PPIE)**  *Desire to reduce acne symptoms and impact* **(QI; PPIE)**  *Recognised benefits of participation* **(TF)** | Initial tailored content suggestions that can be altered at any time  Easily navigable website (iterated with PPI and users)  Weekly SMS/email prompts to return to ACO (including URL) advertising key elements of ACO that may not have been viewed  ‘Progress challenge’ tool provides tailored feedback which congratulates progress and encourages continued engagement and self-monitoring | Physical opportunity  Reflective motivation  Automatic motivation | Environmental context and resources  Goals; Beliefs about capabilities  Emotion; Reinforcement | Environmental restructuring; Persuasion | 2.2 Feedback on behaviour  2.7 Feedback on outcomes of behaviour  7.1 Prompts/cues  10.4 Social reward |
| **Behaviour: consulting a health professional for acne advice and treatment** | | | | | |
| Belief that acne does not warrant medical attention **(QI; TF)**  Concerns about wasting NHS time **(QI)**  Belief that symptoms are not ‘severe’ enough **(QI; TF)** | Advice that acne is a medical condition that does warrant treatment and help seeking, including videos from health professionals:   - encouraging early help seeking - explaining how/when to seek help   Encouragement to consult and seek treatment early to avoid more severe symptoms and/or later scarring  Advice for parents/carers about benefits of early treatment-seeking  Advice addressing common concerns/barriers about consulting (in pharmacy or GP)  Stories from other young people about successful/positive experiences of consulting | Reflective motivation  Automatic motivation | Beliefs about consequences  Emotion | Education; Persuasion; Modelling | 1.2 Problem solving  5.1 Information about health consequences  5.6 Information about emotional consequences  16.3 Vicarious consequences |
| Belief that acne is expected for teenagers and will ‘grow out of it’ **(SR; QI; TF)**  Inaccurate beliefs about causes of acne **(ST; QI; TF; PPIE)** | Information and advice (including videos) about what acne is including about types, causes, and how effective treatments work  Myth-busting about commonly held misconceptions about acne and acne-management | Reflective motivation  Psychological capability | Beliefs about consequences; Optimism  Knowledge | Education; Persuasion | 5.1 Information about health consequences  5.6 Information about emotional consequences |
| Previous negative experiences of consultation **(SR; QI; PPIE)**  Belief that consulting a health professional won’t help **(QI; TF)**  *Belief that they have tried all self-management options available without medical advice* **(ST; QI)**  *Pre-existing positive relationship with HCP/ previous good experience* **(SR; PPIE)** | Information about the various sources of advice and treatments/ consultations options  Advice addressing common concerns/barriers about consulting (in pharmacy or GP)  ‘ChAT guide’ decision aid tool provides tailored output to share with health professional within a consultation to guide discussion and decision making  Stories from other young people about successful/positive experiences of consulting via various routes | Reflective motivation  Social opportunity  Automatic motivation | Beliefs about capabilities; Beliefs about consequences  Social influences  Emotion; Reinforcement | Education; Persuasion; Modelling; Enablement; Environmental restructuring | 1.2 Problem solving  1.4 Action planning  3.2 Social support (practical)  3.3 Social support (emotional)  5.1 Information about health consequences  5.6 Information about emotional consequences  7.1 Prompts/cues  12.5 Adding objects to the environment  16.3 Vicarious consequences |
| Uncertainty about who/where to consult **(QI; PPIE)**  Difficulty making/getting an appointment **(ST; QI; PPIE)** | Practical advice (including video from health professional) about how and when to make an appointment  Information about the various sources of advice and treatments/ consultations options  ‘ChAT guide’ decision aid tool makes tailored recommendations about most appropriate consultation route | Psychological capability  Physical opportunity | Knowledge; Memory attention and decision processes  Environmental context and resources | Education; Enablement; Environmental restructuring | 1.4 Action planning  3.2 Social support (practical)  3.3 Social support (emotional)  4.1 Instruction on how to perform behaviour  5.1 Information about health consequences  5.6 Information about emotional consequences  7.1 Prompts/cues  12.5 Adding objects to the environment |
| Uncertainty/lack of confidence about what to say/ask **(QI; PPIE)**  *Opportunity to prepare for consultation (e.g. make a note of questions)* **(ST)** | ‘ChAT guide’ decision aid tool provides tailored output with key information for health professionals to help guide consultation  Guidance/encouragement videos from health professionals about using the ‘ChAT guide’ when consulting | Psychological capability  Reflective motivation | Knowledge; Memory attention and decision processes  Beliefs about capability | Education; Enablement; Environmental restructuring; Persuasion | 1.4 Action planning  3.2 Social support (practical)  3.3 Social support (emotional)  4.1 Instruction on how to perform behaviour  5.1 Information about health consequences  5.6 Information about emotional consequences  7.1 Prompts/cues  12.5 Adding objects to the environment  15.1 Verbal persuasion about capability |
| Parent/carers don’t wish to consult **(QI:PPIE)**  *Parent/carer leading support-seeking (research, making appointments, asking for advice)* **(ST; QI; PPIE)** | Advice for addressing parental-barriers to help seeking  Advice for parents/carers about benefits of early treatment-seeking and addressing common barriers | Social opportunity | Social influences | Environmental restructuring | 1.2 Problem solving  3.1 Social support (unspecified)  5.1 Information about health consequences  5.6 Information about emotional consequences |
| **Behaviour: Purchasing appropriate non-prescribed treatments** | | | | | |
| Cost of treatments/ financial limitations **(SR; QI; PPIE)** | Advice about prescription exemption eligibility  Advice about pre-payment certificates to help manage costs of prescription treatments  Information about which treatments can be purchased (potentially for less cost) without prescription  Warnings about higher costs of prescribed and non-prescribed treatments if accessed via online pharmacy | Physical opportunity  Psychological capability | Environmental context and resources  Knowledge | Education | 1.2 Problem solving |
| Lack of knowledge/understanding about role of pharmacy **(QI; PPIE)**  Lack of knowledge of where effective non-prescribed treatments can be purchased from **(ST; QI; PPIE)**  *Parent/carer leading support-seeking (research, making appointments, asking for advice)* **(ST; QI; PPIE)** | ‘ChAT guide’ decision aid tool provides tailored recommendations about treatment options and which consultation pathway these can be accessed from  Information about pharmacy and online pharmacy as possible sources of consultation and accessing some treatments  ‘Product review’ tool provides advice on where and how each treatment can be accessed  Stories from other young people about successful/positive experiences of using pharmacy to seek help | Psychological capability  Social opportunity | Knowledge; Memory attention and decision processes  Social influences | Education; Environmental restructuring; Enablement; Modelling | 1.4 Action planning  3.2 Social support (practical)  3.3 Social support (emotional)  4.1 Instruction on how to  perform behaviour  5.1 Information about health consequences  7.1 Prompts/cues  12.5 Adding objects to the environment  16.3 Vicarious consequences |
| Uncertainty about which treatments are most suitable **(QI; PPIE)**  Lack of knowledge about difference between skincare products and evidence-based treatments **(ST; SR; QI; TF; PPIE)** | Advice about difference between proven treatments and skincare products  ‘Product review’ tool facilitates checking of currently used products to see if they include ingredients that actively treat acne  ‘ChAT guide’ decision aid tool provides tailored output about possible suitable treatments based on current symptoms and previously tried treatments, for discussion with health professional  Advice about topical treatments being (usually) first line – including how they work, where to access and how to use | Psychological capability | Knowledge; Memory, attention and decision processes | Education; Environmental restructuring; Enablement | 1.4 Action planning  3.2 Social support (practical)  3.3 Social support (emotional)  4.1 Instruction on how to  perform behaviour  5.1 Information about health consequences  7.1 Prompts/cues  12.5 Adding objects to the environment |
| Belief that treatment will not be effective **(SR; TF)**  Prior experience of purchasing treatments that haven’t worked **(QI; TF; PPIE)**  *Recommendation from a trusted source* **(QI; PPIE; TF)**  *Proven success in someone known to them* **(QI; PPIE)** | Reassurance that symptoms can be improved with consistent use of the right treatments  Advice that wide range of different topical treatments available, and reassurance that there are many topical treatment options to try  Video stories from other young people who have had successful experience with using topical treatments | Reflective motivation  Social opportunity  Automatic motivation | Optimism; Beliefs about consequences  Social influences  Reinforcement | Education; Persuasion; Modelling | 5.1 Information about health consequences  15.1 Verbal persuasion about capability  16.3 Vicarious consequences |
| **Behaviour: instigating use of evidence-based topical treatment** | | | | | |
| Belief that treatment will not be effective **(SR; TF)**  Prior experience of ineffective treatments**(QI; TF; PPIE)**  *Recommended or prescribed by a health professional* **(ST; QI; TF)**  *Desire for sense of control* **(SR;TF)**  *Desire to improve symptoms* **(QI;PPIE)** | Reassurance that a different topical treatment can work even if others have been unsuccessful.  ‘Product review’ tool to help identify comprehensive range of treatment options and provide key information about how to access and use most effectively.  ‘ChAT guide’ decision aid tool provides tailored treatment recommendation options for discussion with heath professional  Advice about difference between proven treatments and skincare products  Advice addressing common concerns about using topical treatments  Video stories from other young people who have had successful experience with using topical treatments | Reflective motivation  Social opportunity  Automatic motivation | Goals; Beliefs about consequences  Social influences  Emotion; Reinforcement | Education; Persuasion; Modelling; Environmental restructuring; Enablement | 1.2 Problem solving  1.4 Action planning  3.2 Social support (practical)  3.3 Social support (emotional)  4.1 Instruction on how to  perform behaviour  5.1 Information about health consequences  7.1 Prompts/cues  12.5 Adding objects to the environment  16.3 Vicarious consequences |
| Concern about experiencing side effects **(ST; TF)** | Detailed tips and strategies for avoiding or minimising side-effects  Video stories from other young people and health professionals about dealing with side effects and strategies to avoid them | Reflective motivation  Automatic motivation | Beliefs about consequences  Emotion | Education; Persuasion; Modelling | 1.2 Problem solving  4.1 Instruction on how to  perform behaviour  16.3 Vicarious consequences |
| Belief that only antibiotics will work **(ST; QI; TF)**  Already using antibiotic or other oral treatment **(QI; PPIE)** | Advice that antibiotics treatment is most effective when accompanied by topical treatment  Explanation about antimicrobial resistance and why important to minimise avoidable (particularly long term) antibiotic use  Information about circumstances when antibiotic treatment might be appropriate | Reflective motivation  Physical opportunity | Beliefs about consequences  Environmental context and resources | Education; Persuasion | 5.1 Information about health consequences  5.3 Information about social and environmental consequences |
| Safety concerns **(ST; TF)** | Reassurance that all topical treatments outlined in Acne Care Online are scientifically tested and proven to be safe and effective  Advice that most topical treatments are safe to use for the majority of people for long-term use  ‘Product review’ tool details specific safety advice relevant to certain medications and circumstances in which it is not safe to use.  Explanation that using topical treatments effectively may avoid use of stronger medications like antibiotics that have more negative side effects. | Psychological capability  Reflective motivation  Automatic motivation | Knowledge; Memory attention and decision processes  Beliefs about consequences  Emotion | Education; Environmental restructuring; Persuasion. | 4.1 Instruction on how to  perform behaviour  5.1 Information about health consequences |
| Belief that an existing skincare product will treat acne **(SR; QI; TF; PPIE)**  Lack of knowledge about difference between skincare products and evidence-based treatments **(ST; SR; QI; TF; PPIE)** | Advice/explanation about difference between proven treatments and skincare products  Searchable ‘Product review’ tool facilitates checking of currently used products to see if they include ingredients that actively treat acne | Reflective motivation  Psychological capability | Optimism; Beliefs about consequences  Knowledge; Memory, attention and decision processes | Education; Environmental restructuring | 5.1 Information about health consequences |
| Lack of understanding of how topical treatments work/unrealistic expectations (**ST; SR; QI; TF; PPIE)** | Explanation (including video) about different types of topical treatments and how they work  Explain that they prevent further spots from forming rather than getting rid of existing spots  Expectation management about how soon topical treatments will work; advice to give them at least 6-8 weeks to start having an effect | Reflective motivation  Psychological capability | Beliefs about consequences  Knowledge | Education | 5.1 Information about health consequences |
| **Behaviour: continued adherence to evidence-based topical treatment** | | | | | |
| No/minimal observable change in symptoms **(SR; QI; TF)**  *Recognised improvement in symptoms* **(SR; QI; TF)**  *Sense of taking control of acne symptoms* **(SR; TF)** | Expectation management about how soon topical treatments will work; advice to give them at least 6-8 weeks to start having an effect  ‘Progress challenge’ tool provides encouraging tailored advice based on how much progress individual reports seeing, and how many weeks they have been using their treatment  ‘Progress challenge’ tool provides weekly reminder to take a progress photo and return to report progress in previous week to reflect on/recognise changes over time  Advice and techniques for maximising effect of treatments such as how to apply and importance of keeping skin moisturised  Video story from young person about how tracking progress helped regular use of treatment | Reflective motivation  Automatic motivation | Beliefs about capabilities; Optimism; Goals; Beliefs about consequences  Emotion; Reinforcement | Education; Persuasion; Modelling; Environmental restructuring | 1.1 Goal setting (behaviour)  1.4 Action planning  1.9 Commitment  2.2 Feedback on behaviour  2.3 Self-monitoring of behaviour  2.4. Self-monitoring of outcomes of behaviour  2.7 Feedback on outcomes of behaviour  4.1 Instruction on how to perform a behaviour  4.2 Information about antecedents  5.1 Information about health consequences  7.1 Prompts/cues  8.3 Habit formation  10.4 Social reward  11.3 Conserving mental resources  15.1 Verbal persuasion about capability  15.3 Focus on past success  16.3 Vicarious consequences |
| Experiencing side effects **(ST; SR; QI; TF; PPIE)** | Detailed tips and strategies for managing most common side-effects  Video stories from other young people and health professionals about dealing with side effects and strategies to avoid them  ‘Progress challenge’ tool provides tailored advice and strategies based on extent to which user reports experiencing side effects | Automatic motivation | Knowledge; Emotion; Reinforcement | Education; Persuasion; Modelling; Environmental restructuring | 2.2 Feedback on behaviour  2.3 Self-monitoring of behaviour  2.4. Self-monitoring of outcomes of behaviour  2.7 Feedback on outcomes of behaviour  4.1 Instruction on how to perform behaviour  4.2 Information about antecedents  5.1 Information about health consequences  15.1 Verbal persuasion about capability  16.3 Vicarious consequences |
| Uncertainty/lack of self-efficacy in applying treatments **(SR; QI; TF)** | Video stories from other young people about successful use of topical treatments  ‘Product review’ tool provides clear instruction about how to apply each treatment  Video demonstrations of topical treatment application  Video advice from dermatologist about applying treatments as part of skincare routine  Initial action planning/ reasons for action planning at start of progress challenge tool to remind users of motivations | Psychological capability  Reflective motivation | Knowledge; Memory, attention and decision processes  Beliefs about capabilities | Education; Environmental restructuring; Training; Persuasion; Modelling | 1.1 Goal setting (behaviour)  1.4 Action planning  1.9 Commitment  4.1 Instruction on how to perform behaviour  6.1 Demonstration of the behaviour  16.3 Vicarious consequences |
| Forgetting to apply **(QI)**  *Having reminders to use treatment regularly* **(QI; PPIE)**  *Having a treatment routine* **(ST)** | ‘Progress challenge’ tool provides tailored advice and strategies about how to build treatment application into daily routines based on how frequently user reports applying in previous week.  ‘Progress challenge’ tool provides weekly reminder to take a progress photo and return to report progress in previous week. | Psychological capability  Reflective motivation  Automatic motivation  Physical opportunity | Memory attention and decision processes; Behavioural regulation  Intentions; Goals  Reinforcement  Environmental context and resources | Environmental restructuring; Education; Persuasion | 1.1 Goal setting (behaviour)  1.4 Action planning  1.9 Commitment  2.2 Feedback on behaviour  2.3 Self-monitoring of behaviour  2.4. Self-monitoring of outcomes of behaviour  2.7 Feedback on outcomes of behaviour  4.2 Information about antecedents  7.1 Prompts/cues  8.3 Habit formation  10.4 Social reward  11.3 Conserving mental resources  15.1 Verbal persuasion about capability  15.3 Focus on past success |
| Being away from home/out of routine **(ST)**  Running out of treatment **(QI)** | ‘Progress challenge’ tool provides tailored advice and strategies about how to plan for being out of routine and ensuring regular supply of treatment | Physical opportunity  Psychological capability | Environmental context and resources  Memory attention and decision processes | Environmental restructuring | 1.4 Action planning  4.2 Information about antecedents  8.3 Habit formation |
| Uncertainty about how long to use/when to stop **(QI; PPIE)** | Reassurance that topical treatments are safe to use long-term  Advice about when/how is most appropriate to use in long term | Psychological capability  Reflective motivation | Knowledge  Beliefs about consequences | Education; Persuasion | 4.1 Instruction on how to  perform behaviour  5.1 Information about health consequences |
| Lack of understanding of how topical treatments work/unrealistic expectations **(ST; SR; QI; TF; PPIE)** | Explanation (including video) about different types of topical treatments and how they work  Explain that they prevent further spots from forming rather than getting rid of existing spots  Expectation management about how soon topical treatments will work; advice to give them at least 6-8 weeks to start having an effect | Reflective motivation  Psychological capability | Beliefs about consequences  Knowledge | Education; Persuasion | 5.1 Information about health consequences  15.1 Verbal persuasion about capability |
| **Behaviour: appropriate skincare for acne-prone skin (alongside effective treatment use)** | | | | | |
| Appearance concerns/desire to cover acne **(ST; SR; QI)**  Desire to wear make-up **(SR; QI)**  Preference/need for close-fitting clothing **(QI)**  Self-consciousness/ embarrassment **(ST; SR; QI; PPIE)**  Picking spots **(ST; QI)**  *Use of acne-safe products* **(ST; QI; PPIE)** | Advice that it’s ok to cover spots if preferred, but reassurance that their skin does not need to look a certain way  Reassurance that acne and spots are very common and many young people will be experiencing similar feelings  Encouragement and advice about use of products (e.g. make up, moisturisers, hair products) that are non-comedogenic  Advice to remove make-up and other cover-up products/items as soon as possible and to ensure skin is cleansed and moisturised  Advice to wear loose fitting breathable material where this covers acne where possible, and/or ensure that skin is cleansed after removal of close-fitting items  Tips/strategies to avoid picking spots, including distraction techniques and managing the physical impact of acne (e.g. how it physically feels)  Advice that picking spots increases risk of later scarring | Reflective motivation  Automatic motivation  Physical opportunity | Social/professional role and identity; Intentions  Emotion  Environmental context and resources | Education; Persuasion; Training; Environmental restructuring | 4.1 Instruction on how to  perform behaviour  5.1 Information about health consequences  5.6 Information about emotional consequences  12.4 Distraction  15.1 Verbal persuasion about capability |
| Lack of knowledge of appropriate skincare routine **(ST; QI)**  *Regular skincare routine* **(QI; PPIE)** | Video advice from Dermatologist about appropriate skincare routine for acne-prone skin  Information/advice about appropriate skincare products to use/avoid in conjunction with evidence based treatments | Psychological capability  Physical opportunity | Knowledge  Environmental context and resources | Education; Training | 4.1 Instruction on how to  perform behaviour  5.1 Information about health consequences  6.1 Demonstration of the behaviour |
| **Behaviour: Utilising strategies to self-manage emotional impact of acne** | | | | | |
| Lack of awareness about/self-efficacy for utilising self-management strategies **(PPIE; QI)** | Step-by step instructions for techniques and exercises to try (including audio recordings):   - Advice/strategies for improving self-perception/ positive self thought - Advice and strategies for remaining positive/hopeful about treatment options - Advice/exercises for relaxation and managing stress - Advice about using existing relationships/building new ones as a source of support | Psychological capability  Reflective motivation | Knowledge; Cognitive and interpersonal skills; Behavioural regulation  Beliefs about capabilities | Education; Training; Enablement | 4.1 Instruction on how to  perform behaviour  5.6 Information about emotional consequences  11.2 Reduce negative emotions |
| Perceived lack of control or powerlessness **(SR; TF)**  *Desire to take control of acne* **(SR; TF)** | Reassurance that there are lots of self-management strategies to help with feeling worried/anxious/upset/stressed/self-conscious about acne  Stories from others about their acne experiences/journey and strategies that have helped them manage their mental health | Automatic motivation  Reflective motivation | Emotion  Beliefs about capabilities; Intentions | Persuasion; Modelling | 5.6 Information about emotional consequences  15.1 Verbal persuasion about capability  16.3 Vicarious consequences |
| **Behaviour:** **Seeking additional external support with emotional impact of acne** | | | | | |
| Lack of awareness of sources of support **(QI; PPIE)** | Reassurance that lots of people will feel worried/anxious/upset/stressed/self-conscious about acne but there are lots of sources of support  Details of useful services/contact details for relevant support services  Advice about overcoming common concerns about seeking support | Psychological capability  Social opportunity | Knowledge  Social influences | Education | 1.2 Problem solving  3.2 Social support (practical)  3.3 Social support (emotional)  4.1 Instruction on how to  perform behaviour  5.6 Information about emotional consequences |
| Concerns that acne isn’t a ‘serious enough’ problem to seek support for **(QI; TF)**  Belief that acne is expected as teenager and that they should be able to manage **(SR; QI; TF)** | Advice that acne is a medical condition that does warrant treatment and help seeking  - including mental health support  Reassurance that lots of people will feel worried/anxious/upset/stressed/self-conscious about acne, but there are lots of things that can help  Encouragement that they should seek support if their acne is bothering them physically or mentally  Advice about preparing for appointments to discuss mental health  Stories from others about their acne experiences/journey and strategies that have helped them manage their mental health | Reflective motivation  Automatic motivation | Beliefs about capabilities  Emotion | Education; Persuasion; Modelling | 4.1 Instruction on how to  perform behaviour  5.6 Information about emotional consequences  11.2 Reduce negative emotions  15.1 Verbal persuasion about capability  16.3 Vicarious consequences |
| Fear/experience of being judged/scrutinised by others **(ST; SR; QI; TF; PPIE)**  Avoidance of social interaction/engagement with others **(ST; SR; QI; PPIE)**  Previous or ongoing difficult/unhelpful existing relationships or interactions **(SR; TF)**  *Connections with others with similar experiences* **(SR; QI; PPIE)**  *Supportive social network* **(QI; PPIE)** | Advice/strategies for dealing with negative/difficult interactions with others/bullying  Advice/strategies for social confidence and connectedness with others  Advice about using existing relationships/building new ones as a source of support  Stories from others about their acne experiences/journey to reassure that they are not alone  Tips for using social media in ways that reduces likelihood of negative mental health impact | Social opportunity  Automatic motivation  Reflective motivation | Social influences  Emotion  Beliefs about consequences | Modelling; Persuasion; Education | 3.2 Social support (practical)  3.3 Social support (emotional)  4.1 Instruction on how to  perform behaviour  11.2 Reduce negative emotions  12.2 Restructuring the social environment  15.1 Verbal persuasion about capability  16.3 Vicarious consequences |

***Sources of evidence: ST** = study team expert opinion; **SR** = systematic review of literature; **QI** = qualitative interview research with young people, parents/carers or health professionals; **TF** = relevant theoretical frameworks/models of behaviour; **PPIE** = Public and Patient Involvement and Engagement

**N.B.** The table provides key examples of evidence about behavioural determinants and intended intervention functions but is not an exhaustive record
