# Supplementary material for: Planning and optimizing a digital self‐management support intervention: Acne Care Online
Source: Br J Health Psychol. 2025 Nov 5;30(4):e70033. doi: 10.1111/bjhp.70033 (PMC12587102; doi:10.1111/bjhp.70033)
Supplement: Supplementary file 2 — File S2. [file BJHP-30-0-s002.docx]

**
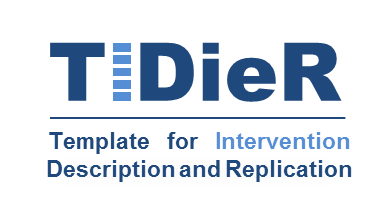
The TIDieR (Template for Intervention Description and Replication) Checklist*:**

Information to include when describing an intervention and the location of the information

| **Item number** | **Item** | **Where located** | |
| --- | --- | --- | --- |
|  |  | Primary paper  (page or appendix  number) | Other (details) |
| **BRIEF NAME** | | | |
| **1.** | Provide the name or a phrase that describes the intervention. | Abstract, p1 |  |
| **WHY** | | | |
| **2.** | Describe any rationale, theory, or goal of the elements essential to the intervention. | Introduction, p2-4 |  |
| **WHAT** | | | |
| **3.** | Materials: Describe any physical or informational materials used in the intervention, including those provided to participants or used in intervention delivery or in training of intervention providers. Provide information on where the materials can be accessed (e.g. online appendix, URL). | ‘The Acne Care Online Intervention’, p22 | (see below for further detail) |
| **4.** | Procedures: Describe each of the procedures, activities, and/or processes used in the intervention, including any enabling or support activities. | ‘The Acne Care Online Intervention’, p22; Behavioural Analysis – Supplementary Table 1; Intervention Logic model, Figure 2 | (see below for further detail) |
| **WHO PROVIDED** | | | |
| **5.** | For each category of intervention provider (e.g. psychologist, nursing assistant), describe their expertise, background and any specific training given. | n/a | (see below for further detail) |
| **HOW** | | | |
| **6.** | Describe the modes of delivery (e.g. face-to-face or by some other mechanism, such as internet or telephone) of the intervention and whether it was provided individually or in a group. | ‘The Acne Care Online Intervention’, p22 | (see below for further detail) |
| **WHERE** | | | |
| **7.** | Describe the type(s) of location(s) where the intervention occurred, including any necessary infrastructure or relevant features. | n/a | (see below for further detail) |
| **WHEN and HOW MUCH** | | | |
| **8.** | Describe the number of times the intervention was delivered and over what period of time including the number of sessions, their schedule, and their duration, intensity or dose. | ‘The Acne Care Online Intervention’, p22 | (see below for further detail) |
| **TAILORING** | | | |
| **9.** | If the intervention was planned to be personalised, titrated or adapted, then describe what, why, when, and how. | ‘The Acne Care Online Intervention’, p22; Optimisation, p20, Table 3 – rationale for tailoring | (see below for further detail) |
| **MODIFICATIONS** | | | |
| **10.** | If the intervention was modified during the course of the study, describe the changes (what, why, when, and how). | Optimisation, p17-21 |  |
| **HOW WELL** | | | |
| **11.** | Planned: If intervention adherence or fidelity was assessed, describe how and by whom, and if any strategies were used to maintain or improve fidelity, describe them. | See below for detail |  |
| **12.** | Actual: If intervention adherence or fidelity was assessed, describe the extent to which the intervention was delivered as planned. | n/a | To be evaluated in ongoing trials |

**Item 3: Materials: Describe any physical or informational materials used in the intervention, including those provided to participants or used in intervention delivery or in training of intervention providers. Provide information on where the materials can be accessed (e.g. online appendix, URL).**

The intervention is delivered entirely via a web-app accessible from any internet connected device.

**Item 4:** **Procedures: Describe each of the procedures, activities, and/or processes used in the intervention, including any enabling or support activities.**

All procedures, activities and processes are delivered via the Acne Care Online digital intervention with any ‘offline’ behaviour self-initiated by the user if and

when they choose to do so. Regular (once per week for first 6 weeks, then fortnightly until 12 weeks) SMS/email prompts encourage them to return to Acne Care Online periodically to engage with content. During the study period, users are also prompted (via email/SMS) to return to the online platform to complete online measures every 12 weeks for 12 months (4 separate occasions). If participants do not complete online measures, they will be contacted by telephone by the study team to complete the measures over the phone.

**Item 5: For each category of intervention provider (e.g. psychologist, nursing assistant), describe their expertise, background and any specific training given.**

Not applicable – standalone digital intervention, but see ‘Study team knowledge and expertise’, p8 for detail of expertise involved in development of digital intervention content

**Item 6: Describe the modes of delivery (e.g. face-to-face or by some other mechanism, such as internet or telephone) of the intervention and whether it was provided individually or in a group.**

Standalone digital intervention provided on individual basis - accessed via a link from recruitment materials (after online consent, screening, baseline measures and randomisation). Optimised for use on mobile phone, but accessible from any internet connected device

**Item 7: Describe the type(s) of location(s) where the intervention occurred, including any necessary infrastructure or relevant features.**

Standalone digital intervention optimised for use on mobile phone, but accessible from any internet connected device. Participants can access anytime/place with access to a device and connection to the internet

**Item 8: Describe the number of times the intervention was delivered and over what period of time including the number of sessions, their schedule, and their duration, intensity or dose.**

As soon as participants are randomised to the intervention group, they have immediate access to the intervention for the whole 12 month study period. Their access is entirely self-directed and they can access as often as they would like. Regular (once per week for first 6 weeks, then fortnightly until 12 weeks) SMS/email prompts encourage them to return to Acne Care Online periodically to engage with further content. Each time they return, after logging in they return to their ‘dashboard’ where they see their tailored recommendations for relevant content sections. They can choose to view any of these, or to explore any of the rest of the Acne Care Online content from the ‘homepage’.

**Item 9: If the intervention was planned to be personalised, titrated or adapted, then describe what, why, when, and how.**

On initial login, individuals’ responses to two tailoring questions about any current treatments, and their priorities for advice and support are used to make personalised recommendations about specific sections that are likely to be most relevant. These appear on their ‘dashboard’ – the page they return to on each subsequent login to Acne Care Online. However, users can still opt to access any content beyond these recommendations at any time. Should their preferences change in terms of their priorities for support, they can also choose to re-answer the initial tailoring questions at any time to provide updated recommendations. This feature aims to provide optional structure/guidance about which content to look at, in which order for those who want this, without being prescriptive about all users having to click through compulsory content that some may not find helpful.

The ‘ChAT guide’ decision aid tool also provides personalised recommendations about treatment options and consultation advice based on participant answers to questions about: the perceived severity of their symptoms, treatments they have already tried, where on their body their acne is, and how their acne affects them. These responses then determine the tailored feedback they receive, which is delivered in a format that can be taken to/shared with health professionals during/ in advance of a consultation. Intervention users can access and use this feature as often as they would like. It aims to facilitate effective discussion with consultations and shared/informed decision making about treatment options that are relevant for the individuals circumstances.

The progress challenge tool offers weekly tailored feedback on participants own self-monitoring of their behaviour and outcomes for the preceding week. Those who sign up to the progress challenge set an initial action plan documenting which topical treatment(s) they are using, and how often they plan to apply them. They then receive a weekly SMS/email reminder to return to record how often they used their treatment, the extent to which they experienced side effects, and to what extent they are happy (or otherwise) with their progress. Based on their responses and how many weeks into the challenge they are (1-6), they are provided with tailored recommendations about how to procced in the following week. If they are progressing well with few issues, they are likely to be congratulated on progressed and encouraged to keep going. If progress is less positive, and/or they are experiencing challenges they will be provided with appropriate recommendations, tips and strategies and reassurance and encouragement where appropriate.

**Item 11: Planned: If intervention adherence or fidelity was assessed, describe how and by whom, and if any strategies were used to maintain or improve fidelity, describe them.**

Engagement with the digital Acne Care Online content will be monitored (and later analysed) using participant level usage data recorded by the software used to build and host the intervention. This will help to answer questions about how levels of engagement/dose relate to intervention outcomes and/or participant characteristics. Quantitative and qualitative process evaluation will also seek to assess engagement and adherence, with quantitative process measures included that collect data about adherence to acne medications, and qualitative process interviews to explore and understand participants engagement with/adherence to both online and offline behaviours.

Regular (once per week for first 6 weeks, then fortnightly until 12 weeks) SMS/email prompts encourage them to return to Acne Care Online periodically to engage with further content
